# A personal history of research on hypertension From an encounter with hypertension to the development of hypertension practice based on out-of-clinic blood pressure measurements

**DOI:** 10.1038/s41440-022-01011-1

**Published:** 2022-09-08

**Authors:** Yutaka Imai

**Affiliations:** 1 Tohoku Institute for the Management of Blood Pressure, Sendai, Japan; 2 Emeritus Professor Tohoku University, Sendai, Japan

**Keywords:** Home blood pressure, Ohasama study, HOMED-BP study, Blood pressure variability, The Japanese Society of Hypertension Guidelines

## Abstract

In the 1970s, many people had severe hypertension and related cardiovascular and cerebrovascular diseases; however, antihypertensive treatments were not available at the time. The author encountered such conditions during the initial exposure to medicine. The author subsequently entered the field of hypertension medicine to prevent such conditions and engaged in hypertension research for more than 50 years. The author’s central interest was the physiological and clinical aspects of blood pressure (BP) variability. Out-of-clinic BP measurements were the focus of clinical research. It was anticipated that self-measurement of BP at home (HBP) would improve medical practice surrounding hypertension. To establish evidence-based hypertension medicine, the Ohasama study (an epidemiology based on HBP) was conducted. The study provided firm evidence of the clinical significance of HBP and diagnostic criteria for hypertension and normotension. To establish target HBP levels for antihypertensive therapy, the Hypertension Objective treatment based on Measurement by Electrical Devices of Blood Pressure (HOMED-BP) study (a prospective intervention study) was also conducted. Application of HBP measurements expanded to obstetric, clinical pharmacology, pathophysiology, and genetic studies. During these studies, crucial information on the clinical significance of BP variability (such as circadian and day-by-day variation of BP, nocturnal BP, white-coat hypertension, and masked hypertension) was established. Finally, the author described the priority of HBP over clinic-measured BP for the diagnosis of hypertension in the 2014 Japanese Society of Hypertension Guidelines. In this article, the author’s history of hypertension research, from the first encounter with hypertension to the construction of guidelines on hypertension, is reviewed.

## Introduction

In October 2019, I received a prestigious award from the Japanese Society of Hypertension. This honor was awarded for the successful completion of the Ohasama Study, Hypertension Objective treatment based on Measurement by Electrical Devices of Blood Pressure study (the HOMED-BP study), and other studies on clinical hypertension based on out-of-clinic blood pressure (BP) measurements. Professor Kazuomi Kario, the Editor in Chief of Hypertension Research, invited me to contribute a life-work review, that is, my personal history of hypertension research, including the Ohasama Study and the HOMED-BP study. He suggested that this paper would encourage young researchers of hypertension and offer a guide for research in the field of clinical hypertension. I took him up on his kind offer. I ask that readers assess this article not as a scientific review but as a monologue and medical essay on my personal journey of hypertension research. Therefore, I appreciate your understanding, as I have referred only to my own research and have not mentioned preceding or simultaneously performed crucial research conducted worldwide.

## Personal history of hypertension research

### Encounter with hypertension

I was born in 1946 in Maebashi City, Gunma Prefecture, Japan. My mother died from a hypertensive disorder of pregnancy at the age of 36 years when I was just 8 years of age. She suffered from malignant hypertension and renal failure. At that time, there was no available antihypertensive treatment. My father was a doctor of internal medicine, but he just looked on with folded arms. This might have been the formative experience and trauma that motivated me to engage in the world of hypertension research.

### Age of junior residents

I graduated from Tohoku University School of Medicine in 1971 and spent two and a half years as a junior resident at Yuri General Hospital, Honjo, Akita Prefecture. At that time, the Yuri-Honjo area had the highest incidence of hypertensive cerebral bleeding in Japan. Patients with severe hypertension were common. Dr. Shojiro Izumi, Director; Dr. Masashi Itoh, Associate Director, and many medical staff of the Yuri General Hospital, pushed forward with practice prevention and epidemiological surveys of hypertension, in which I also participated. I am surprised that self-measurement of BP using a mercury sphygmomanometer and stethoscope had already been introduced in their epidemiological surveys.

At that time, there was no effective treatment for hypertension, especially severe hypertension. Many hypertensive patients died of stroke, congestive heart failure, and renal insufficiency. I had the option of a path as a neurologist. However, after encountering the serious and miserable situations of young to middle-aged patients who suffered from irreversible conditions, I chose a path to prevent stroke, that is, hypertension medicine.

### Age of postgraduate school

In 1974, I was admitted to the Second Department of Medicine, Tohoku University School of Medicine, chaired by Professor Kaoru Yoshinaga. Because I was interested in circulatory regulation, he dispatched me to the Second Department of Pharmacology, chaired by Prof. Norio Taira, who was an expert and authority in cardiovascular pharmacology. He provided me with a theme regarding venous return. This task helped me notice that BP had phasic and tonic regulatory mechanisms [1]. This sparked my interest in BP variability. I began my clinical research in 1976 with the supervision of Prof. Kaoru Yoshinaga. He assigned me a task related to the clinical pharmacology of diuretics. I performed self-measurement of BP at home (home BP [HBP]) using a semiautomatic aneroid sphygmomanometer as a tool for clinical pharmacology. This experience persuaded me to apply HBP as a tool for research in the clinical science of hypertension based on its outstanding characteristics, particularly high measurement frequency, unified measurement conditions and high reproducibility of the measurement value.

### Before and after study abroad

In 1984, I was invited by Prof. Colin Johnston, Department of Medicine, Monash University, Melbourne. Based on my experience in the Department of Pharmacology in Sendai, I started my experiment on cardiovascular autonomic control. Since Prof. Colin Johnston kept Brattleboro rats, hereditary vasopressin-deficient rats, I used these rats to examine the effect of vasopressin on baroreflex control of BP. Baroreflex sensitivity decreased in vasopressin-deficient rats through attenuation of vagal function, which led to high BP variability [2]. After returning to Japan, I continued my experiments using Brattleboro rats and found that centrally administered vasopressin induced BP stabilization through facilitation of the parasympathetic nerve system [3]. Furthermore, centrally administered vasopressin induced hypotensive and bradycardic effects in spontaneously hypertensive rats [4].

### Circadian BP variation and nocturnal BP

Based on these experimental results, BP variability has become the focus of attention in hypertension research and practice. Circadian BP variation is an important factor that affects BP variation. At that time, devices for ambulatory BP monitoring (ABPM) using an upper arm cuff were introduced in Japan. However, these devices were too large, heavy, and noisy. Intermittent cuff inflation significantly disturbed sleep. To overcome these shortcomings of conventional ABPM devices, we developed a finger volume-oscillometric device capable of performing ABPM [5]. Using this device, circadian BP variations in several forms of secondary hypertension were studied. The most important finding of these studies was the loss or reversal of normal nocturnal dipping of BP and high nocturnal BP in Cushing’s syndrome [6]. The effect of excess glucocorticoids on circadian BP variation was subsequently confirmed by the effect of exogenously administered glucocorticoids [7]. These variations in circadian BP were later labeled ‘nondipper’ and ‘inverted dipper (riser)’, which led to hypertensive cardiovascular target organ damage and events. These studies were presented at the 12^th^ Annual Meeting of the International Society of Hypertension, Kyoto, in 1988. I concluded that advanced cardiovascular damage in Cushing’s syndrome might be partly due to nocturnal hypertension. This was the beginning of our focus on nocturnal BP. Simultaneously, I observed and reported that high BP in the morning (morning hypertension) was accompanied by nocturnal hypertension as well as nondipping. Although it was a later development, I explored the possibility of measuring nocturnal BP using an HBP measurement (HBPM) device, which led to the publication “Device for the self-measurement of BP that can monitor BP during sleep” in 2001 [8]. Furthermore, this exploration progressed to the development of a “home nocturnal BP monitoring system using a wrist-cuff device” in 2018 that did not disturb nocturnal sleep [9]. In addition to Cushing’s syndrome, I studied circadian BP variation in essential hypertension, pheochromocytoma, primary aldosteronism, hyperthyroidism, renovascular hypertension, renal insufficiency, preeclampsia, and autonomic failure. These observations were reported in the State-of-the-Art lecture at the 13th Annual Meeting of the International Society of Hypertension, Montreal in 1990 [10]. This transmission of information across the world brought about communication among international researchers in the field of BP measurements. Later, this research group held the 8th International Consensus Conference on Blood Pressure Monitoring in Sendai in 2001 (Supplementary Fig. 1).

### The Ohasama study

Through my experience over 15 years of engaging in clinical science and practice of hypertension, I noticed the clinical significance of HBPM and ABPM. However, around the 1980s, the clinical significance of HBP received little attention. Professor Shigeaki Hinohara, a legendary doctor of clinical medicine in Japan, often told us that when he started his research on HBP in the 1970s, the Ministry of Health and Welfare, Japan, warned him not to make patients self-monitor their BP at home. They told him that BP measurements are medical practice and that patients should not measure their BP by themselves. Around the same time, I started to measure HBP in the general population and in hypertension clinics. At that time, many physicians told me that patients should not measure their own BP at home, and many study participants reported to me that they were scolded by family doctors, “Don’t measure BP by yourself at home”.

Almost 30 years ago, I contributed to a paper on HBP in the American Heart Association Journal. One of the reviewers commented, “What is home BP? Home has no BP”, indicating very low awareness of HBPM in that era.

In 1986, Dr. Kenichi Nagai, Director of Ohasama Prefectural Hospital, Ohasama, Iwate prefecture, who was my classmate at Tohoku University School of Medicine, asked me how to increase health awareness and preventive awareness regarding hypertension among Ohasama inhabitants. I suggested that he make the Ohasama inhabitants measure HBP. Obtaining HBPM devices and encouraging them to measure HBP was the challenge. Nothing ventured, nothing gained. I called a person responsible for the marketing of Omron Life Science Co. LTD　(Present Omron Health care Co. Ltd) and asked him to support our project. He offered us 300 semiautomatic electrical sphygmomanometer devices (Omron HEM401C).

At that time, Ohasama Town had a population of 8000 people. The movement of people in Ohasama Town was small. The Ohasama Prefectural Hospital was the sole medical institute in this area. Young and active public health nurses and the highly conscious local administration of Ohasama encouraged us. Furthermore, Ohasama inhabitants had a strong interest in HBPM, since at that time, they were aware that the stroke incidence in Ohasama was very high and that hypertension was an important cause of stroke.

In 1987, the HBPM devices were distributed to each household. Each member of the household over 12 years of age measured their own BP and recorded it in a log book. The inhabitants of Ohasama continued their BP measurements for 35 years, and their outcomes were followed up over these periods. Simultaneously, ABPM was initiated. However, ABPM was aborted after 12 years because it was too burdensome for public health nurses to visit and apply devices to each individual. ABPM received unfavorable criticism from the inhabitants. These findings suggest that ABPM is difficult to introduce widely into general clinical practice. In contrast, the Ohasama Study shed light on the high feasibility and utility of HBPM.

### First in the world achievements from the Ohasama study

The prognostic significance of ambulatory blood pressure (ABP) was first reported by Dr. Dorothee Perloff (San Francisco, CA, USA) and colleagues in 1983 through a prospective analysis of 1076 hypertensive patients.

The Tecumseh Blood Pressure Study in Michigan, USA, was a pioneering epidemiological survey of BP based on HBPM. Although the Tecumseh Blood Pressure Study started in the 1970s, HBPM was introduced in the 1980s, and participants were followed up for three years. The first observation was reported in 1990 by Dr. AD Mejia and colleagues as ‘normative data on BP self-determination’. This study included normotensive subjects aged 18-42 years old. The Ohasama Study started in 1986 and followed up with the participants for 35 years. The Ohasama Study included the general population of Ohasama aged between 7 and 98 years, and the normative value of HBP was first reported in 1993 [11],; thus, the following results reported from the Ohasama Study were largely the first epidemiological findings of HBP.

### Reference values of HBP and ABP

In 1993, the reference values of HBP and ABP were first reported in the Ohasama Study [11, 12]. These were the results of the cross-sectional analysis.

Around that time, I did not have the ability to analyze prospective data statistically. However, young and talented people were recruited for the Ohasama Study projects; these members are shown in Supplementary Table 1. Many doctoral and postdoctoral fellows from the Department of Clinical Pharmacology, Tohoku University, which I have chaired since 1999, have joined this project.

The first and most important results of the Ohasama Study were reported in 1997 [13], followed by a report in 1998 [14]. In the latter study, 1491 individuals aged ≥40 years in Ohasama without a history of stroke were followed up for an average of 10.6 years. HBP could be used to predict stroke incidence, whereas conventional BP values obtained during health examinations could not. HBP ≥ 135/85 mmHg significantly increased the relative hazard of stroke incidence (Supplementary [15] Fig. 2), and these values were referred to as the hypertensive level. These reports were cited in The Sixth Report of the Joint National Committee, 1997, USA; World Health Organization-International Society of Hypertension Guideline, 1999; European Society of Hypertension/European Society of Cardiology Guidelines for Management of Arterial Hypertension 2007 and 2013; British Guidelines for management of Hypertension 2011; and Japanese Society of Hypertension Guidelines 2004, 2014 and 2019. Thereafter, a normal BP level of <125/85 mmHg was reported in the Ohasama Study. These values are now the basis of reference values for hypertension and normotension based on HBP in the International Hypertension Guidelines. Thus, the Ohasama Study has been recognized worldwide.

### Barriers to implementation of the Ohasama Study

During the 35 years of the Ohasama Study, we faced several crises related to the continuation of the study. The largest one was a 1999 press report by a major newspaper that reported that the Ohasama Study analyzed genes without permission from study participants. This was the top newspaper article. Certain unfavorable persons treated me like a criminal, while many colleagues defended me. We obtained informed consent from the residents after explaining that their remaining blood samples were stored for future research. However, the explanation sheets did not include the term “gene”. At that time, there were no official ethical guidelines for genetic research in Japan; thus, the need for specific informed consent for genetic research was not introduced. Many newspaper reporters visited Ohasama for follow-up coverage. Many residents of Ohasama told reporters, “We acknowledge the researchers of the Ohasama Study since they are supporting our health. We received an explanation of their genetic analysis”.

Thus, the Ohasama Study was saved and continues to date. I thank the many supporters and residents of Ohasama from the bottom of my heart.

### Differential prognostic significance among ABP, HBP and clinical BP

Finally, we wanted to determine which BPM, HBP, ABP, or clinical BP (CBP) provided the most useful information for the diagnosis and treatment of hypertension. Few studies have directly compared the usefulness of ABP, HBP, and CBP. In 2005, Dr. Robert Sega (Milano, Italy) and colleagues first reported from the Pressioni Arteriose Monitorate eLovo Associazione (PAMELA) study that the risk of death increased with a given increase in HBP or ABP rather than CBP, but the overall ability to predict death was not greater for HBP and ABP than for CBP. In the PAMELA study, the average of only two HBP measurements was used. In the Ohasama Study, 49 HBP measurements were averaged over four weeks. As a result, the clinical indications for ABPM and HBPM overlapped, and the clinical significance of each method for predicting target organ damage might differ for different organs [16].

### Contribution to international and domestic joint research

Data on HBP and ABP were provided for international and domestic joint research. I greatly appreciate Prof. Jan Staessen (Leuven, Belgium), who organized the International Database of Ambulatory Blood Pressure in relation to Cardiovascular Outcome (IDACO) (Supplementary references 1) and the International Database of Home Blood Pressure in relation to Cardiovascular Outcome (IDHOCO) meta-analyses (Supplementary references 2). These meta-analyses established firm evidence for the clinical significance of HBP and ABP. I acknowledge that the results of the IDACO and IDHOCO meta-analyses corroborated many results of the Ohasama Study. The Asia Pacific Cohort Studies Collaboration (APCSC) (Supplementary references 3), Blood Pressure Lowering Treatment Trialist’s Collaboration (BPLTTC) (Supplementary references 4), ambulatory blood pressure international (ABP-International) study (Supplementary references 5), prospective study collaboration (Supplementary references 6), Chronic Kidney Disease Prognosis Consortium (Supplementary references 7), Evidence for Cardiovascular Prevention from Observational Cohort in Japan (EPOCH-JAPAN) (Supplementary references 8), and the Japan Arteriosclerosis Longitudinal Study (JALS) (Supplementary reference 9) are international and domestic joint studies. I am proud that the Ohasama Study could contribute to such global efforts.

### White-coat hypertension and masked hypertension

Pressor response and hypertension in response to the medical environment have been well recognized for a long time. Prof. Thomas G Pickering (New York, USA) termed this “white-coat hypertension” in 1988. The diagnosis of white-coat hypertension cannot be discussed without BP measurement outside the clinic.

Whether white-coat hypertension is harmful or innocent remains unclear. The Ohasama Study demonstrated that white-coat hypertension was a transitional condition to hypertension, suggesting that white coat hypertension carried a poor cardiovascular prognosis [17]. In fact, we demonstrated in a meta-analysis of four cohorts that white-coat hypertension was not a benign condition for stroke in the long term [18].

In 1996, we reported a prognostic significance for mortality among white-coat and “reverse white-coat hypertension” [19]. The term “reverse white-coat hypertension” was later referred to as “masked hypertension” by Prof. Thomas G Pickering in 2002.

The term “masked hypertension” is unmatched when compared to “reverse white-coat hypertension.” The Ohasama Study first demonstrated that masked hypertension was associated with a high risk for all-cause mortality [19] and cardiovascular/stroke mortality [20]. We also demonstrated first that the risk of silent cerebrovascular lesions is higher with masked hypertension than with sustained normotension [21]. In an international hypertension meeting, Professor Thomas G Pickering mentioned that the Ohasama Study first reported a high risk of masked hypertension. I was impressed with his sense of fairness.

The diagnoses of white-coat hypertension and masked hypertension are diverse based on the differential out-of-clinic BP measurements. The impact of partial white-coat hypertension (either home or ambulatory normotension with clinical hypertension) and partially masked hypertension (either home or ambulatory hypertension with clinical normotension) is comparable to that of completely masked hypertension (both home and ambulatory hypertension with office normotension) or sustained hypertension [22].

The Japan Home versus Office Measurement Evaluation (J-HOME) study first reported the prevalence and predictive factors of treated white-coat hypertension and treated masked hypertension in a real-world larger-scale observational study [23]. Similar survey results are listed in Supplementary references 10.

### Circadian BP variation, nocturnal BP and morning BP in the Ohasama Study

Prof. Eoin O’Brien (Dublin, Ireland) and colleagues first noticed in 1988 that people with nondipping nocturnal BP, nondippers, have a higher risk of cerebrovascular complications than people with normal circadian BP variation, dippers. In 1990, Prof. Kazuyuki Shimada (Kouchi, Japan) and colleagues reported the risk of nocturnal hypertension for silent cerebrovascular disease. Around the same time, I reported disturbed circadian variation in BP in several forms of secondary hypertension [6, 7, 10], that is, nondipping and inverted dipping of nocturnal BP. Therefore, the investigation of circadian and nocturnal BP variations was of urgent interest in the Ohasama Study. The first report from the Ohasama Study was focused on the relationship between nocturnal BP and silent cerebrovascular lesions in elderly subjects [24]. We observed that in women with multiple lacunar infarctions, the amplitude of the nocturnal fall of systolic BP was greater than that in women without lacunar infarction [24]. The greater amplitude of nocturnal fall of BP was a real finding. This apparently excessive fall in nocturnal BP might be equivalent to the term “extreme dipper”. Nocturnal hypotension has long been considered the “sine qua non” of extreme dippers. First, we concluded that excessive falls in nocturnal BP and resulting inappropriately low nocturnal BP levels were responsible for the risk of silent cerebrovascular lesions. However, in our analysis, we did not adjust the amplitude of the nocturnal BP fall to the nocturnal BP level as well as the daytime BP level. The daytime and nocturnal BP levels in subjects with lacunar infarction were higher than those in subjects without lacunar infarction [24].

The following results from the Ohasama Study demonstrated that extreme dipping was a benign condition when compared to nondipping and inverted dipping [25]. The mortality risk was highest among inverted dippers, followed by nondippers; there was no difference in mortality between extreme dippers and dippers [25].

#### Nondipper and inverted dipper

A very high risk for mortality of inverted dippers was first reported epidemiologically from the Ohasama Study [25]. We also reported that a diminished nocturnal fall in BP was a risk factor for cardiovascular mortality independent of the overall BP load during a 24-h period in the general population [26]. However, it is also logically derived that nocturnal BP levels in nondippers with 24-h normotension are relatively higher than those in dippers with 24-h normotension.

#### Extreme dipper

**Fig. 1:**
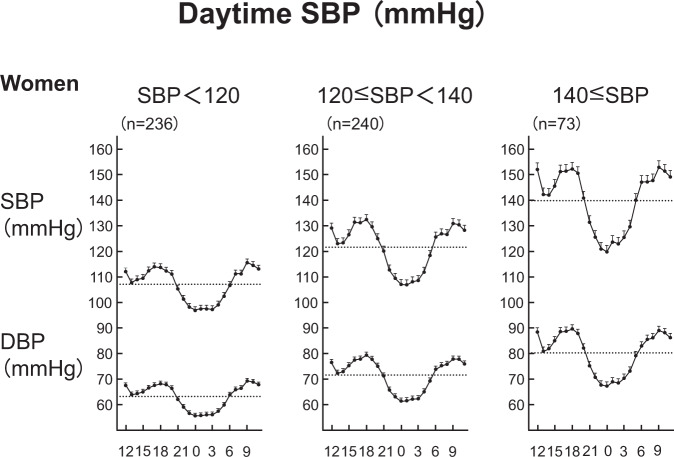
Circadian blood pressure variation among women with different daytime systolic blood pressure (SBP) levels in the Ohasama study. As daytime ambulatory blood pressure levels increased, the amplitude of nocturnal blood pressure decrease increased. A substantial number of subjects with high daytime blood pressure had an extreme dipping pattern of circadian blood pressure variation, while nocturnal blood pressure levels were hypertensive

Looking back on this experience, we epidemiologically reported that women with daytime systolic BP ≥140 mmHg and extreme dipping had higher nocturnal BP than those with daytime systolic BP <120 mmHg and normal dipping [27] (Supplementary Fig. 3). When daytime systolic BP was high, nocturnal BP was also high, even when these participants had extreme dipping (Fig. 1). In other words, the risk of extreme dipping behavior in hypertensive subjects was not necessarily due to an excessive nocturnal fall of BP/excessively low nocturnal BP levels but might be, at least in part, due to a high level of either or both nocturnal and daytime BP.

#### Nocturnal hypertension

Assessing this phenomenon, it is true that nondippers and inverted dippers seem to have a high risk of mortality and morbidity from cardio- and cerebrovascular diseases. It is also true that high nocturnal BP levels might be responsible for the high risk of mortality and morbidity associated with cardio- and cerebrovascular diseases. Even in extreme dippers with daytime hypertension, high nocturnal BP levels might partly be responsible for their high risk. The high predictability of cardio- and cerebrovascular risk of nocturnal BP was first reported in the systolic hypertension in Europe (Syst-Eur) study by Prof. Jan Staessen and others in 1999. The Ohasama Study clearly demonstrated that the total cardio- and cerebrovascular mortality risk was significantly associated with elevated nocturnal systolic BP [28, 29]. The Ohasama Study was the main source of the IDACO database, which demonstrated the high prognostic accuracy of nighttime ABP [30, 31].

#### Morning BP

The remaining topics of circadian BP variation were morning BP, morning surges, and morning hypertension. Looking back, a motive for why I drew attention to the circadian variation of BP in Cushing’s syndrome was very high morning BP just after awaking in patients with this syndrome [6]. We were the first to observe differential characteristics of morning and evening HBP. In subjects in the Ohasama Study, the morning HBP was higher than the evening HBP [32]. Interestingly, the use of antihypertensive medication in the morning was positively associated with the difference between morning HBP and evening HBP, indicating that an insufficient duration of action of antihypertensive drugs contributed to the high morning BP [33]. This observation led me to introduce HBP as a tool for assessing the clinical pharmacology of antihypertensive drugs.

In 2001, we reported that the risk of cardiovascular mortality was higher in subjects with large positive differences between morning and evening HBP than in subjects with small or negative differences, indicating that when morning HBP was relatively or absolutely higher than evening HBP, the risk of cardiovascular mortality was high [34]. We also demonstrated that morning hypertension determined by HBPM, which indicates hypertension specifically observed in the morning, was a good predictor of stroke, particularly among individuals using antihypertensive medication [35], suggesting again that insufficient duration of antihypertensive drug action was responsible for the morning hypertension [34]. Finally, we reported that risk stratification in international hypertension guidelines has stronger predictive power when based on morning HBP than on clinic BP [36, 37].

#### Morning surge

A remarkable rise in BP in the early morning has been referred to as the morning surge. Many historical studies have indicated that cardio- and cerebrovascular events occur more frequently in the early morning, when the morning surge of BP develops. A pioneering study by Dr. Iwao Kuwajima (Tokyo, Japan) in 1995 reported that the magnitude of the morning surge in BP after rising from bed was related to the severity of hypertensive target organ damage. Thereafter, the risk of morning surge for cardio- and cerebrovascular diseases has been widely reported. However, the risk of morning surge differs among definitions, age, race, and pathophysiological conditions of the disease. For example, in the Ohasama Study, morning surge was not associated with a risk of cerebral infarction, but an increased risk of cerebral hemorrhage was observed in subjects with a large morning pressor surge [38].

The IDACO meta-analysis, which included data from the Ohasama Study, demonstrated that only an extreme morning surge conveyed a risk for cardiovascular events [39]. The mechanical and dynamic impact of extreme morning surges on vasculature may cause cardiovascular events. We have shown that the product of the rate of rise of morning surge (dp/dt, the slope of morning rise of BP) and amplitude of morning surge (ΔBP) are independent risk factors for predicting cardiovascular events and stroke [40].

**Fig. 2:**
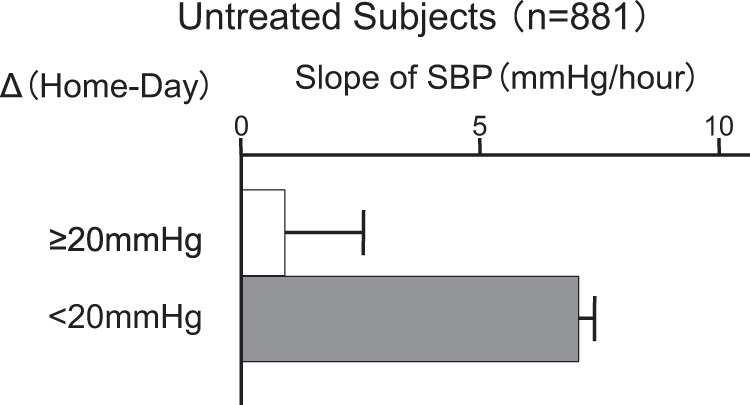
Relation between slopes of morning pressor response seen from preawakening to postawakening period and the difference between morning blood pressure levels in subjects treated with antihypertensive drugs in the Ohasama study. Subjects with a steep slope of morning pressor response, such as extreme dippers, had relatively low morning blood pressure when compared to daytime ambulatory blood pressure. In other words, morning hypertension is rather rare in extreme dippers

A remarkable rise in BP in the early morning, the morning surge, may produce a large difference between morning BP level and nocturnal BP level; thus, subjects with a morning surge may have low nocturnal BP and/or high morning BP, and therefore, morning surge should occur in dippers, including extreme dippers and/or subjects with morning hypertension. The Ohasama Study demonstrated that dippers and extreme dippers had a lower risk than nondippers and inverted dippers [25, 26]. The morning surge should have a steep slope of the pressor phenomenon from nighttime during sleep to the morning after rising. However, the Ohasama Study demonstrated that high morning BP relative to average daytime BP occurred in subjects with a loose or negative slope of the pressor phenomenon, indicating that high morning BP was observed in nondippers and inverted dippers. In contrast, in subjects with a steep slope of the pressor phenomenon, dippers and extreme dippers had minimal morning high BP (Fig. 2). These observations suggest that the risk of morning hypertension reflects the risk of nondippers and/or inverted dippers. Furthermore, nondippers and inverted dippers had relatively or absolutely high nocturnal BP.

There are many concepts related to the risk of cardio- and cerebrovascular disease in relation to pathological circadian BP variation, such as nondipper, inverted dipper, extreme dipper, morning surge, and morning hypertension. What is the principle of the unification of these concepts? Considering the results of the Ohasama Study in a comprehensive way, high nocturnal BP is postulated to be a common risk factor for mortality and morbidity of cardio- and cerebrovascular diseases among pathological circadian BP variations.

#### Clinical significance of differential BP indices

Many previous studies have demonstrated the clinical significance of isolated systolic hypertension, isolated diastolic hypertension, pulse pressure, and double products. We also focused on these indices based on HBP and ABP epidemiologically. Thus, our findings are novel in this field. The risk of isolated systolic hypertension assessed by HBP had a significantly higher relative hazard than that of normotension, while isolated diastolic hypertension carries a low risk of cerebrovascular mortality, similar to that found in subjects with normotension [41]. This result was later confirmed by the IDACO meta-analysis [42]. In contrast, the Ohasama Study demonstrated that pulse pressure assessed by ABPM was a weak predictor of stroke when compared to systolic ABP [43]. Finally, we found that the double product (product of home systolic BP and home pulse rate) had a predictive value for mortality [44].

### BP variability

My motivation to research hypertension started with my interest in BP variability. Physical materials are relatively resistant to continuous stress levels but are more susceptible to intermittent stress. Therefore, it might be expected that individuals with an increased lability of BP would suffer from more cardiovascular diseases.

#### Short-term BP variation

To the best of our knowledge, the relationship between short-term variability (every 30 min) and target organ damage was first reported in 1987 by Prof. Gianfranco Parati (Milano, Italy) and colleagues. However, this result was derived from a cross-sectional analysis, and the cause-effect relationship between BP variability and target organ damage was unclear. The prognostic significance of short-term BP variability was first reported in the Ohasama Study [45]. The BP and heart rate variabilities obtained every 30 min by ABPM were independent predictors of cardiovascular mortality in the general population. We subsequently reported that ABP variability was closely associated with carotid artery alterations [46]. This result was later confirmed by the IDACO meta-analysis [47].

#### Day-by-day variation of HBP

We then focused on mid-term BP variability, that is, the day-by-day variability of HBP. Since the reproducibility of ABP and CBP is poor, the reproducibility of short-term BP variability and visit-to-visit BP variability are also poor; thus, these parameters might be unstable indices for BP variability. Because the reproducibility of HBP is high [48, 49], day-by-day variability of HBP might be a good index of BP variability. The Ohasama Study first reported that day-by-day variability of HBP was independently associated with increased risks for cardiovascular and stroke mortality [50]. Later, the IDHOCO meta-analysis confirmed the results of the Ohasama Study [51]. The result that day-by-day variability in HBP was associated with cognitive decline was a novel finding from the Ohasama Study [52]. However, the Ohasama Study also reported that although day-by-day variability of HBP predicted cardiovascular mortality, this index of BP variability did not incrementally predict outcomes over and beyond mean systolic BP [53]. This may be partly because factors such as older age, female sex, elevated HBP, low home heart rate (HR), and elevated home HR variability are significant determinants of elevated day-by-day variability of HBP [54, 55].

#### Seasonal variation

Seasonal variations in BP are another important component of BP variability. HBPM is the most appropriate method for assessing seasonal variation in BP. Seasonal variation in BP determined by HBPM was first assessed in 1996 [56]. We emphasized the importance of environmental temperature and daytime length for seasonal variation in BP [56]. We also reported that the small-to-middle seasonal variation in HBP, which may be partially attributed to earlier seasonal adjustment of antihypertensive medication, was associated with better cardiovascular outcomes [57].

### HR based on HBP and ABP

A priority of the Ohasama Study is not only HBP and ABP but also HR obtained from HBPM and ABPM. The prognostic value of resting HR obtained in a clinical setting has been widely recognized. Several previous studies, including the Ohasama Study, demonstrated that nocturnal HR predicted mortality [58, 59]. The Ohasama Study first focused on short-term HR variability and found that the cardiovascular mortality rate increased linearly with a decrease in daytime HR variability [45]. Furthermore, high daytime systolic BP variability together with low daytime HR variability carried an extremely high risk of cardiovascular mortality [45]. The ABP data of the Ohasama Study were included in the ABP-International meta-analysis and demonstrated that ambulatory HR during sleep strongly predicted cardiovascular risk [60].

However, the clinical significance of home HR has not received much attention. The Ohasama Study demonstrated that an increase in morning home HR was associated with an increase in the risk of cardiovascular mortality [61]. In contrast with short-term HR variability, an increase in home HR variability was also associated with cardiovascular mortality [50]. We concluded that short-term HR variability might reflect baroreflex function, while elevated day-by-day variability of home HR might reflect activation of the sympathetic nervous system [50].

### Application of strict and reliable phenotypes of HBP and ABP for hypertension research

#### Application of HBP and ABP to clinical pharmacology

HBPM is useful for assessing the response to antihypertensive medication. The effectiveness of HBPM in evaluating the effect of antihypertensive drugs can be explained by the absence of digit preference, observer bias, and white-coat reaction. However, most of the beneficial effects are caused by the increased number of measurements under unified measurement conditions for long periods. These factors improve the reproducibility of BP measurement. Furthermore, minimal placebo and regression to the mean effect were observed for HBP. These characteristics of HBP make it most appropriate as a tool for the clinical pharmacology of antihypertensive drugs [62]. HBPM can detect minimal antihypertensive effects using a minimal number of subjects [62]. In clinical studies, we examined the effect of guanabenz, imidapril, perindopril, valsartan, losartan, indapamide, bisoprolol, amlodipine, doxazosin, spironolactone, eplerenone, and others. These results have been reported elsewhere (Supplementary references 11). HBP is useful not only to evaluate the antihypertensive effect of drugs but also to estimate the duration of action of antihypertensive drugs. The duration of action of antihypertensive drugs was estimated by the trough-to-peak ratio (T/P ratio) of antihypertensive drugs based on ABPM. However, since the reproducibility of ABP is poor, the reproducibility and reliability of the T/P ratios are also poor. We introduced HBP to estimate the duration of action of antihypertensive drugs, that is, the morning effect to evening effect ratio (M/E ratio) [63]. Thereafter, the time to attain the maximum antihypertensive effect was examined using exponential decay function analysis. This analytical method was first reported by Dr. Keiichi Mashima (Okayama, Japan) and colleagues and is a very sophisticated method. We applied this method to examine the BP lowering effect and the time to attain the maximum antihypertensive effect (stabilization time) of 7 angiotensin II receptor blockers (ARBs). We demonstrated that the maximum effect and stabilization time among ARBs were extremely diverse and emphasized that an ARB should be chosen based on its desired characteristics [64, 65]. Thus, HBPM is now an essential tool for evaluating the effect of antihypertensive drugs.

### HOMED-BP study and telemedicine

The Ohasama Study and many clinical studies on hypertension on the basis of out-of-clinic BP measurements provided us with reference values of hypertension and normotension of HBP and informed us of the superior characteristics of HBP for clinical hypertension. The next step was to determine the target BP levels for antihypertensive drug treatment.

**Fig. 3:**
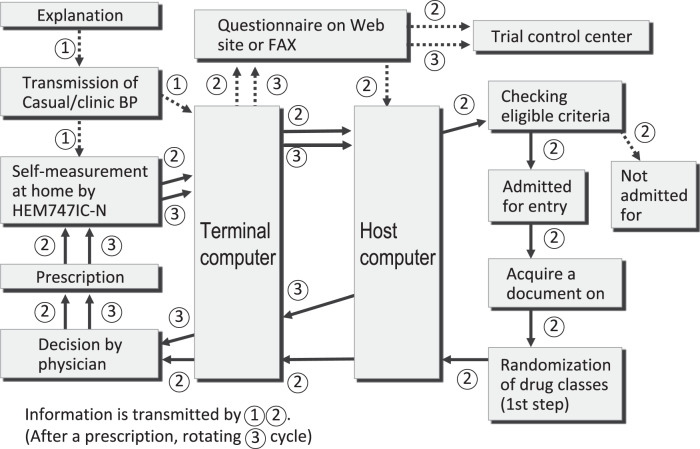
Flow chart of registration and drug prescription through the internet for the HOMED-BP study

To adequately introduce HBP into clinical practice, target BP levels for antihypertensive treatment needed to be determined. A prospective intervention study was indispensable to establish the target BP levels. One intervention study, the Treatment of Hypertension Based on Home or Office Blood Pressure (THOP) trial, was implemented and reported in 2004 by Prof. Jan Staessen and colleagues before our study. The THOP trial reported the cost-effectiveness of HBP in comparison to CBP but did not refer to target BP levels of HBP. The HOMED-BP trial started to register untreated low-risk essential hypertensive subjects in 2001 and involved a total of 3518 patients who were randomly assigned in a 2 × 3 design to usual control (125–134/80–84 mmHg) vs. tight control (<125/<80 mmHg) groups and to the initiation of drug treatment with three randomly assigned classes of antihypertensive drugs: angiotensin-converting enzyme inhibitors (ACE-I), ARBs, or calcium channel blockers (CCB). The HBP was measured using integrated circuit memory equipment. After the transmission of saved HBP values to the terminal microcomputer, the stored data were transmitted to the host computer at every visit to the clinic through the internet. Based on these HBP values, the host computer randomized the first-line drugs as well as the target BP levels. The host computer also determined the necessity for additional therapy or dose titration in further steps to reach the randomized target BP. The host computer sent the prescription to the practitioners’ terminal computers. The HOMED-BP study was the first prospective randomized controlled trial of antihypertensive drug therapy that adopted a telemedicine system (Fig. 3). Because the target HBP level of tight control was too low to follow for practitioners at that time, the HBP level in the tight control group was not different from that in the usual control group; thus, no significant difference in outcome was observed between the two groups. Thus, we analyzed the data observationally using all the data from all subjects. Upon follow-up, the systolic HBP, which revealed a 1% 5-year risk of the primary outcome, was approximately 130 mmHg [66]. The HOMED-BP study demonstrated that antihypertensive treatment even in low-risk, mild hypertension decreases cardiovascular events dramatically when strict control of HBP to 130 mmHg or less has been achieved. This result was reported in 2012 [66]. The cardiovascular risk for patients with HBP levels less than 135/85 mmHg was significantly lower than the risk for patients with HBP over 135/85 mmHg in nondiabetic subjects. This study also confirmed the high feasibility, utility, practicability, and reliability of telemedicine in antihypertensive treatment. In the subanalysis of the HOMED-BP study, target HBP levels in patients with impaired glucose metabolism were set at less than 125/75 mmHg [67]. These values were cited as target HBP levels in the Japanese Society of Hypertension Guidelines 2019.

As very important information from the HOMED-BP study, we analyzed the factor of Wilder’s law, that is, the proportional antihypertensive drug effect according to the baseline pretreatment home BP level. We initially hypothesized that Wilder’s law is mediated by the white-coat effect accompanied by regression to the mean effect. It has been confirmed that HBP is not impacted by the white-coat effect or regression to the mean effect. However, the HOMED-BP study demonstrated that Wilder’s law persisted even in HBP measurements, suggesting that regression to the mean phenomenon did not contribute to this law. In other words, the phenomenon observed according to Wilder’s law in HBP is physiological [68].

Thus far, intervention trials for antihypertensive drugs have been based on the promotion of pharmaceutical companies. The HOMED-BP study was the first physician-led clinical trial based on the teletransmission of HBP. Almost 500 physicians voluntarily participated in the HOMED-BP study. There was no reward for the registration of patients, implementation of the trial, or report generation. Nevertheless, 500 participants continued the trial, in which HBP data were transmitted through the internet every month for an average of 7 years, indicating that HBPM was practiced without difficulty and was readily accepted by practitioners and patients. An incentive for them was the development of a new approach to the diagnosis and treatment of hypertension. I still have a fond memory of Prof. Kim Sam-Soo, Seoul, Korea making a statement at the Japanese Society of Hypertension Meeting that it may be impossible to implement such a study in Korea; such a study could only be implemented because of Japanese national traits. I was very proud that we were able to complete the study. At present, it seems difficult to implement intervention trials of antihypertensive drugs since pharmaceutical companies no longer support research of this nature. Therefore, the results of observational studies such as the Ohasama Study, the HOMED-BP study, and the IDHOCO meta-analysis may replace the results of an intervention trial. In fact, the American College of Cardiology/American Heart Association (ACC/AHA) guidelines 2017 took notice of the results of observational studies such as the prospective study collaboration as a target BP of antihypertensive therapy.

### Effect of environment and lifestyle on HBP

Since HBPM and ABPM can detect small changes in BP, we applied HBPM to evaluate the effects of environmental, internal, and lifestyle factors on BP in the Ohasama Study. Habitual and heavy alcohol intake was associated with higher morning HBP [69]. In active smokers, systolic HBP levels and day-by-day variability of HBP were associated with cerebral infarction [70]. Surprisingly, even passive smoking was associated with high HBP [71]. We observed an acute pressor phenomenon in HBPM just after the Great East Japan Earthquake of 2011 [72]. High-level consumption of fruits, vegetables, potassium, and vitamin C was associated with a significantly lower risk of HBP hypertension [73] and a lower risk of developing future HBP hypertension [74]. The Ohasama Study found that parental premature death was significantly associated with higher HBP levels in adult offspring [75].

### Research on the relationship between pathophysiological condition and out-of-clinic BP

The final goal of the Ohasama and HOMED-BP studies was to prevent cardiovascular diseases, including renal disease. In the Ohasama Study, we focused on indices of atherosclerosis such as carotid intima-media thickness, carotid plaque, pulse wave velocity, augmentation index, central BP, arterial stiffness index, pulse pressure, and brain magnetic resonance imaging in relation to out-of-clinic BP measurement. The results of these studies are shown elsewhere (Supplementary references 12).

The clinical significance of out-of-clinic BP measurement was clarified based on the relationship between out-of-clinic BP measurement and stroke incidence in the Ohasama Study. In the Ohasama Study, the role of out-of-clinic BP measurement mainly focused on the risk of stroke incidence as stroke was still the main cause of cardiovascular mortality and morbidity in the Ohasama area. Many publications on the relationship between out-of-clinic BP measurement and stroke incidence are listed in Supplementary references 13.

Chronic kidney disease was also a main target of out-of-clinic BP measurement as a cause and effect of hypertension in the Ohasama Study. Several approaches to this pathological condition have been performed using out-of-clinic BP measurement (Supplementary references 14). I believe that these results pioneered epidemiologic studies based on out-of-clinic BP measurements.

The characteristics of out-of-clinic BP measurement, such as high measurement frequency and high reproducibility, result in highly reliable data and high sensitivity to changes in BP in response to physiological stimuli. Using this advantage of out-of-clinic BP measurement, we examined the role of biological indices, such as the renin-angiotensin-aldosterone system, in BP regulation (Supplementary references 15).

### Genetic study in the Ohasama Study and the HOMED-BP study

At the start of the Ohasama Study, genetic studies were in the process of development. Fortunately, researchers at Osaka University showed an interest in the Ohasama Study. Prof. Toshio Ogihara, Prof. Hiromi Rakugi, Prof. Tomohiro Katsuya, and Prof. Kei Kamide are the main members of the study group at Osaka University, and several achievements have been published (Supplementary reference 16). In my opinion, the study result demonstrating that the accumulation of common polymorphisms associated with the development of hypertension determined by HBPM was the most impressive result from the Ohasama Study. The study included only 403 Ohasama inhabitants. A strict and reliable phenotype obtained by HBPM may contribute to high sensitivity for genotyping [76]. The genome-wide association study assessing the response to antihypertensive medication was examined using HBPM in the HOMED-BP study. Several single nuclear polymorphisms have been associated with responses to CCBs and ARBs. The approach based on high-fidelity phenotyping by HBP might be a step toward personalized treatment of hypertension [77].

## The Babies’ and their parents’ longitudinal Observation in Suzuki Memorial Hospital on Intrauterine period (BOSHI) study

**Fig. 4:**
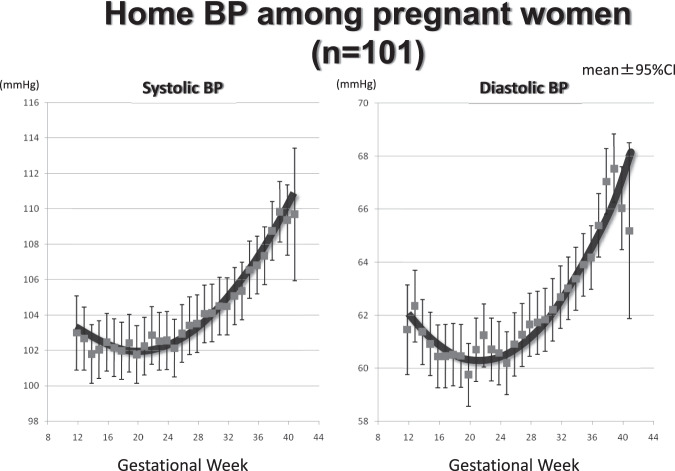
Trends in home blood pressure levels during pregnancy among 122 normal pregnant women

As mentioned at the beginning of this article, I have described my personal history of an encounter in my childhood with a hypertensive disorder of pregnancy. I thought that at some point, I would like to research hypertensive disorders during pregnancy. That opportunity came when I met Dr. Masakuni Suzuki, a renowned gynecologist/obstetrician, an emeritus professor at Tohoku University and director of Suzuki Memorial Hospital, Iwanuma, Japan. We introduced HBPM to the medical examinations of pregnant women as a part of the BOSHI study. BOSHI means “mother and child.” The trend in HBP levels during pregnancy among 122 normal pregnant women clearly demonstrated a mid-pregnancy fall. The curve of HBP values with advancing gestational age was J-shaped. We demonstrated a normal range of HBP at each gestational age (Fig. 4). Using these data, we could define a hypertensive disorder of pregnancy from the very early stages of pregnancy [78]. We found that the trends in HBP during pregnancy were extremely different among those who delivered in different seasons [79].

We studied the relationship between BP during pregnancy in pregnant women and BP during pregnancy in the mothers of pregnant women. Pregnant subjects with higher maternal BP during pregnancy had a higher BP throughout pregnancy than those with lower maternal BP. The mid-pregnancy fall in BP disappeared in the former group [80] (Supplementary Fig. 6).

The relationship between prepregnancy body mass index (BMI) and HBP during pregnancy was also examined. Obese pregnant women have higher HBP throughout pregnancy. It was also noted that the mid-pregnancy fall in BP declined or disappeared in obese pregnant women. It was concluded that the control of prepregnancy weight might be important for the management of BP during pregnancy [81] (Supplementary Fig. 7).

We further observed a postpartum BP surge, which was strictly defined by HBPM. During the third trimester, BP gradually increased and reached its highest level on the day of delivery. After delivery, the HBP values increased rapidly. The peak HBP was observed from days 6 to 8 after delivery and was elevated by 7 mmHg of systolic BP even in normal pregnant women, namely, a postpartum BP surge [82] (Supplementary Fig. 8). Since cerebrovascular events are frequently observed during the postpartum period and the concept of postpartum eclampsia has been recognized, HBP should be monitored not only during pregnancy but also during the postpartum period. Mid-pregnancy falls and the associated HBP level and trajectory of HBP during pregnancy were strongly associated with infant birth weight [79, 83]. These results were truly novel findings since HBPM was first introduced in these obstetric studies. These pregnancy-related small changes in BP are detected only by HBPM. Finally, in recent years, obstetricians have paid attention to mild elevation in BP during early pregnancy and cardiovascular disease risks such as obesity, dyslipidemia, impaired glucose metabolism, high normal BP, and genetic predisposition as risk factors for hypertension disorder of pregnancy/preeclampsia in the prepregnancy period. I would like to ensure that women of child-bearing age monitor HBP before becoming pregnant.

### Benefit of HBPM and medical economics of HBPM

In Japan, HBP devices have been purchased by the general population and have been used by most hypertensive patients. From the Ohasama Study and National Statistics of Japan, it was calculated that the introduction of HBPM in the care of hypertension would result in a decrease in annual medical expenditure of approximately 1 trillion yen, that is, 100 billion US dollars [84, 85]. Such economic benefits of HBPM are derived from its superiority over ABPM and CBPM.

We have reviewed the superiority of HBPM over ABPM and CBPM elsewhere [86, 87]. HBPM offers good daily and long-term repeatability and trackability of measurements and has no regression to the mean and placebo effects. However, ABPM is unsuitable for repeated measurements. ABPM performed less well than HBPM, largely due to the high cost and heavy burden on medical staff and patients. Furthermore, ABPM is not easily available or feasible. Low utility, tolerability, and acceptability of ABPM by patients are apparent.

**Fig. 5:**
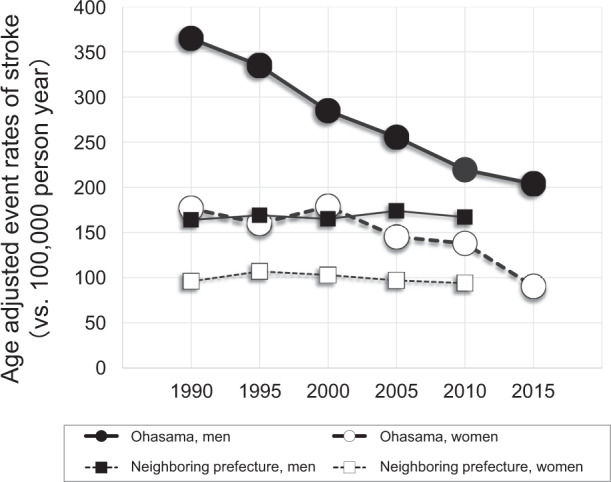
Trends in event rates of stroke in Ohasama and the neighboring prefecture from the time close to the start of the Ohasama study to 2015

HBPM is the most effective way to monitor ongoing diseases in a timely manner. This method can provide direct feedback for the diagnosis of hypertension, white-coat hypertension, masked hypertension, and BP control. A specific feature of HBPM is that it is available not only for hypertensive populations but also for nonhypertensive populations. It is assumed that monitoring HBP in nonhypertensive and hypertensive populations increases awareness of BP and has an impact on the motivation for lifestyle modification. HBPM mediates patients’ active participation in hypertension management and improves adherence to antihypertensive medications. Additionally, HBPM activates doctors’ motivation for active treatment, overcomes doctors’ therapeutic inertia, and facilitates doctor‒patient cooperation. These characteristics of HBPM improve BP control and decrease cardiovascular risk, leading to beneficial effects in medical economics. After the introduction of HBPM in Ohasama, stroke event rates decreased dramatically over 20 years, especially in men, when compared to the neighboring prefecture (Fig. 5).

The introduction of HBPM in the management of hypertension allows health care professionals to capture BP data from patients without frequent visits to the clinic, with the potential to curtail the escalating cost of health care. Although the cost-effectiveness of HBPM is high, administrative authorities in Japan do not approve reimbursement for the diagnosis and treatment of hypertension based on HBPM. Coverage of health insurance and reimbursement for HBPM may be the most effective way to promote the widespread use of HBPM for physicians and patients. Public education on hypertension is important for the acceptance of HBPM in the general population.

### Contribution to the Japanese society of hypertension guidelines

As a worldwide first, we developed guidelines for HBPM in 2003 [88] and 2012 [89]. The Japanese Society of Hypertension Guidelines 2014 [90] and 2019 [91] proposed a flowchart for the diagnosis of hypertension to help individuals without a history of hypertension recognize the presence of hypertension in a health check or a self-measurement of BP and consult a clinic. Clinical BP is measured in the clinic. Simultaneously, HBP measured by patients is reported to clinicians or patients begin to measure HBP before the start of treatment according to physicians’ recommendations. Since criteria for the diagnosis of hypertension based on HBP have been established, the diagnosis of hypertension is based on patients’ CBP and HBP levels.

In this case, when there is a discrepancy in decisions between the two methods, HBP-based diagnosis of hypertension is prioritized because the prognostic value of HBP is higher than that of CBP, and HBPM can rule out white-coat hypertension and masked hypertension. Thus, the Japanese Society of Hypertension asserted that HBPM has priority in the diagnosis and treatment of hypertension and is indispensable for the practice of hypertension management. We have included this flowchart in the 2014 Japanese Society of Hypertension Guidelines. I wrote the chapter on the measurement and clinical evaluation of BP as a writing member of the Japanese Society of Hypertension Guidelines. First, I expected that many committee members would reject such a proposal. Surprisingly, no one opposed this proposal. The reason why all Japanese Society of Hypertension committee members agreed with this proposal might be based on the situation of the practice of hypertension in Japan. For example, as a major premise, 40 million devices for HBPM have already been distributed in Japan. In fact, British guidelines still emphasize that if the CBP is 140/90 mmHg or higher, ABPM but not HBPM should be offered to all patients to confirm the diagnosis of hypertension. However, is this possible in real-world practice? Personal communication with an expert on hypertension in England suggests that the application of ABPM systems to patients with suspected hypertension is rare.

Historically, only ABPM has been recommended as an out-of-clinic BP measurement method in the US and European guidelines for the diagnosis and treatment of hypertension. Recently, the US guidelines 2017 and the European guidelines 2018 accepted our initiative, although they did not cite the 2014 Japanese guidelines. The US and European guidelines state that almost all patients with suspected hypertension must undergo HBP or ABP measurement to diagnose true hypertension and rule out white-coat hypertension and masked hypertension. A few years ago, a person responsible for the ACC/AHA guideline committee visited Japan. At that time, I asked him whether ABPM was widely applied in US clinical practice. He told me no. Thus, even in the United States, HBP is mainly used for an out-of-clinic BPM.

### Future perspective on out-of-clinic BP measurements

When we started work on out-of-clinic BP measurements, ABPM received high evaluation and attracted full attention as a tool for hypertension research. HBPM has received little attention given the auxiliary role of ABPM and its supporting role in the management of hypertension. However, I anticipated that HBPM would be the leading action for the diagnosis, treatment, and study of hypertension. In addition to hypertension medicine, HBPM should be applied in cardiovascular, renal, obstetrics, pediatric, geriatric, and neurology medicine. In particular, the introduction of HBPM into the obstetric field may markedly improve the outcomes of pregnancy. The introduction of HBPM to cardiovascular preventive medicine might improve accuracy in the assessment of cardiovascular risk factors. The introduction of HBPM to health education at school might improve health consciousness in younger generations.

Tele-transmission of HBP has great potential not only for medical practice but also for preventive medicine and welfare. I planned to apply HBPM to the remote health care system in the Ohasama area. Ohasama was absorbed and merged with Hanamaki in 2005. The population of Ohasama in 1985 was 8053, and that of the Ohasama area of Hanamaki City is now less than 5000. The rate of people aged over 65 years old in Ohasama in 1985 was 16.2%; this has now risen to nearly 50%. The Ohasama Prefectural Hospital, which had 50 beds, was eliminated and converted to a nonbed clinic. Furthermore, inconvenient transportation makes it difficult for elderly inhabitants of the Ohasama area to access medical care. Thus, the medical environment in the Ohasama area continues to deteriorate. In this medical environment, I considered the necessity of preventive interventions. I established the Tohoku Institute for the Management of Blood Pressure, where I developed a remote health management system using telemonitoring of HBP, which was first implemented in the Ohasama area. The system is very effective for the health care and welfare of Ohasama inhabitants and is useful for connecting Ohasama inhabitants with medical institutions. This system might prevent the isolation of elderly persons living alone.

Telemonitoring of HBP may improve the quality of practice in hypertension management. The condition of hypertension is underdiagnosed and undertreated worldwide. Several factors are involved in these conditions, such as low adherence to antihypertensive drugs, skepticism about hypertension medicine, low literacy on hypertension and its therapy in patients as well as practitioners, and high economic burden, although therapeutic inertia of the practitioners might be the major cause. It has been confirmed that HBPM has a favorable impact on these conditions. We developed a medical support system using HBP teletransmission, which instructs prescriptions to obtain target HBP levels according to patients’ pathophysiological conditions. I expect that this system will improve practitioners’ therapeutic inertia.

## Conclusion

When I started my research on BP measurements, I did not expect that the work would continue for more than 35 years. A series of coincidences made it possible for the work to continue, but this was not intended. Many fortunes and tiny misfortunes made it possible to continue work. On some occasions, it was “good that comes out of evil”. The title of my retirement commemoration publication was “Michi-naki-mich”, which implies “uncharted path”.

The best of luck was encounters with many coworkers and colleagues all over Japan, Ohasama residents, Ohasama government officers, health promotion staff, medical technology companies and pharmaceutical companies, and many friends in the academic circle. Many domestic and global pioneering researchers have encouraged me and supported this work.

I want to finish the manuscript with immense gratitude for all of them.

## Supplementary information

Supplementary Table 1

Supplementary Figure 1

Supplementary references
