# Supplementary material for: A personal history of research on hypertension From an encounter with hypertension to the development of hypertension practice based on out-of-clinic blood pressure measurements
Source: Hypertens Res. 2022 Sep 8;45(11):1726–42. doi: 10.1038/s41440-022-01011-1 (PMC9637554; doi:10.1038/s41440-022-01011-1)
Supplement: Supplementary file 1 — Supplementary Table 1 [file 41440_2022_1011_MOESM1_ESM.doc]

**Supplementary Table 1**

Collegues who are/were active in academic circle

Prof. Takayoshi Ohkubo, Teikyo University

Prof. Atsushi Hozawa, Tohoku University

Prof. Masahiro Kikuya, Teikyo University

Prof. Kei Asayama, Teikyo University

Prof. Hirohito Metoki, Tohoku Medical and Pharmaceutical University

Prof. Kazuhito Totsune, Tohoku Fukushi University

Prof. Takao Sakamoto, Tohoku University

Ptof.Kennichi Chonan, Showa Pharmaceutical University

Prof. Fumika Yamamoto, Tokyo City Univercity

Associate Prof. Ryusuke Inoue, Tohoku University

Associate Prof. Azusa Hara, Keio University

Associate Prof. Taku Obara, Tohoku University

Associate Prof. Keiko Hosohata, Osaka Medical and
Pharmaceutical University

Associate Prof. Masako Fujiwara, Tohoku University

Senior Associate Prof. Megumi Tsubota-Utsugi, Teikyo University

Assistant Prof. Takahisa Murakami, Tohoku Medical and Pharmaceutical University

Assiatant Prof. Takuo Hirose, Tohoku University

Assiatant Prof. Michihiro Satoh, Tohoku Medical and
Pharmaceutical University
