# Supplementary material for: A personal history of research on hypertension From an encounter with hypertension to the development of hypertension practice based on out-of-clinic blood pressure measurements
Source: Hypertens Res. 2022 Sep 8;45(11):1726–42. doi: 10.1038/s41440-022-01011-1 (PMC9637554; doi:10.1038/s41440-022-01011-1)
Supplement: Supplementary file 2 — Supplementary Figure 1 [file 41440_2022_1011_MOESM2_ESM.pptx]

## Slide 1
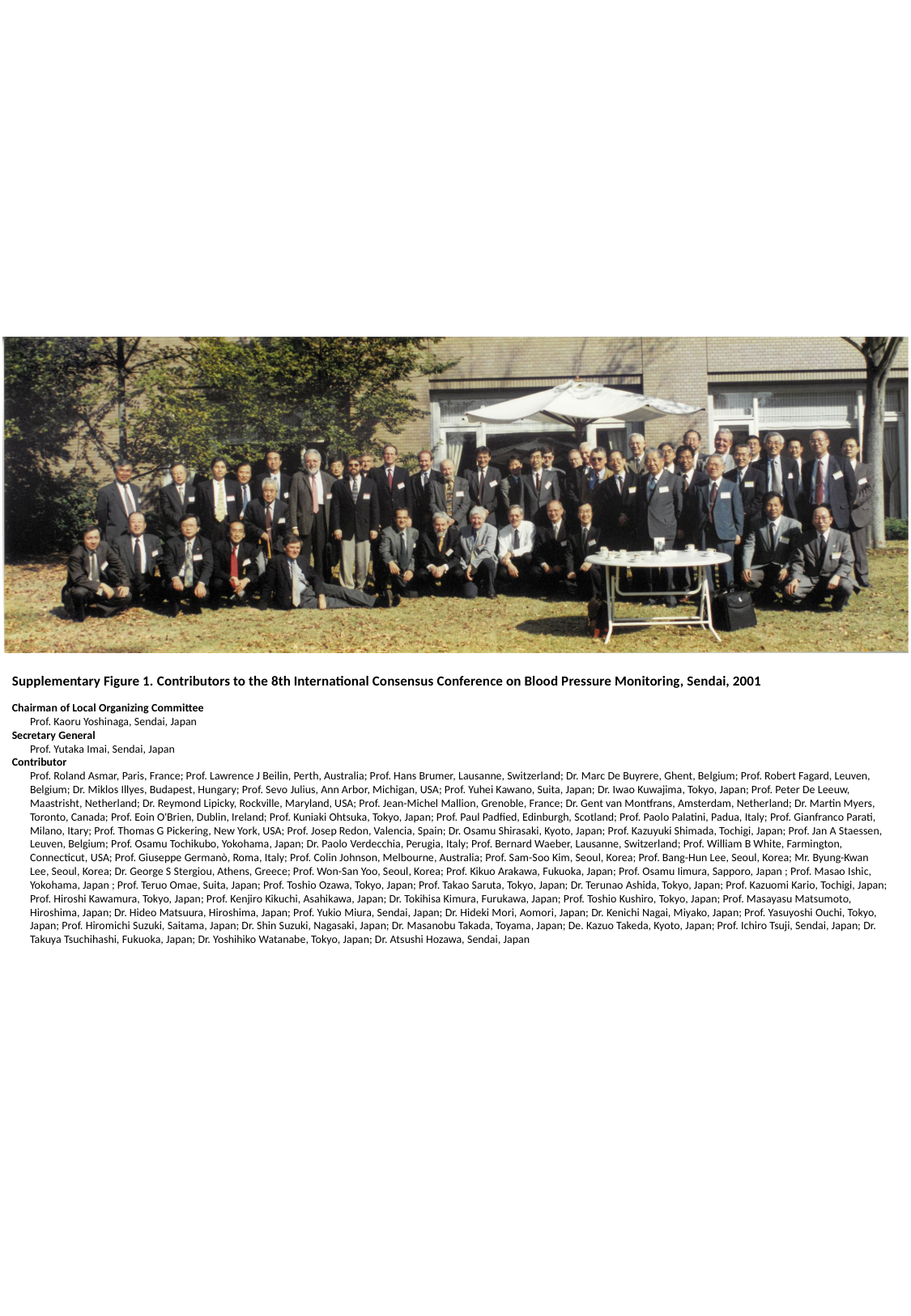

Supplementary Figure 1. Contributors to the 8th International Consensus Conference on Blood Pressure Monitoring, Sendai, 2001
Chairman of Local Organizing Committee
Prof. Kaoru Yoshinaga, Sendai, Japan
Secretary General
Prof. Yutaka Imai, Sendai, Japan
Contributor
Prof. Roland Asmar, Paris, France; Prof. Lawrence J Beilin, Perth, Australia; Prof. Hans Brumer, Lausanne, Switzerland; Dr. Marc De Buyrere, Ghent, Belgium; Prof. Robert Fagard, Leuven, Belgium; Dr. Miklos Illyes, Budapest, Hungary; Prof. Sevo Julius, Ann Arbor, Michigan, USA; Prof. Yuhei Kawano, Suita, Japan; Dr. Iwao Kuwajima, Tokyo, Japan; Prof. Peter De Leeuw, Maastrisht, Netherland; Dr. Reymond Lipicky, Rockville, Maryland, USA; Prof. Jean-Michel Mallion, Grenoble, France; Dr. Gent van Montfrans, Amsterdam, Netherland; Dr. Martin Myers, Toronto, Canada; Prof. Eoin O'Brien, Dublin, Ireland; Prof. Kuniaki Ohtsuka, Tokyo, Japan; Prof. Paul Padfied, Edinburgh, Scotland; Prof. Paolo Palatini, Padua, Italy; Prof. Gianfranco Parati, Milano, Itary; Prof. Thomas G Pickering, New York, USA; Prof. Josep Redon, Valencia, Spain; Dr. Osamu Shirasaki, Kyoto, Japan; Prof. Kazuyuki Shimada, Tochigi, Japan; Prof. Jan A Staessen, Leuven, Belgium; Prof. Osamu Tochikubo, Yokohama, Japan; Dr. Paolo Verdecchia, Perugia, Italy; Prof. Bernard Waeber, Lausanne, Switzerland; Prof. William B White, Farmington, Connecticut, USA; Prof. Giuseppe Germanò, Roma, Italy; Prof. Colin Johnson, Melbourne, Australia; Prof. Sam-Soo Kim, Seoul, Korea; Prof. Bang-Hun Lee, Seoul, Korea; Mr. Byung-Kwan Lee, Seoul, Korea; Dr. George S Stergiou, Athens, Greece; Prof. Won-San Yoo, Seoul, Korea; Prof. Kikuo Arakawa, Fukuoka, Japan; Prof. Osamu Iimura, Sapporo, Japan ; Prof. Masao Ishic, Yokohama, Japan ; Prof. Teruo Omae, Suita, Japan; Prof. Toshio Ozawa, Tokyo, Japan; Prof. Takao Saruta, Tokyo, Japan; Dr. Terunao Ashida, Tokyo, Japan; Prof. Kazuomi Kario, Tochigi, Japan; Prof. Hiroshi Kawamura, Tokyo, Japan; Prof. Kenjiro Kikuchi, Asahikawa, Japan; Dr. Tokihisa Kimura, Furukawa, Japan; Prof. Toshio Kushiro, Tokyo, Japan; Prof. Masayasu Matsumoto, Hiroshima, Japan; Dr. Hideo Matsuura, Hiroshima, Japan; Prof. Yukio Miura, Sendai, Japan; Dr. Hideki Mori, Aomori, Japan; Dr. Kenichi Nagai, Miyako, Japan; Prof. Yasuyoshi Ouchi, Tokyo, Japan; Prof. Hiromichi Suzuki, Saitama, Japan; Dr. Shin Suzuki, Nagasaki, Japan; Dr. Masanobu Takada, Toyama, Japan; De. Kazuo Takeda, Kyoto, Japan; Prof. Ichiro Tsuji, Sendai, Japan; Dr. Takuya Tsuchihashi, Fukuoka, Japan; Dr. Yoshihiko Watanabe, Tokyo, Japan; Dr. Atsushi Hozawa, Sendai, Japan

## Slide 2
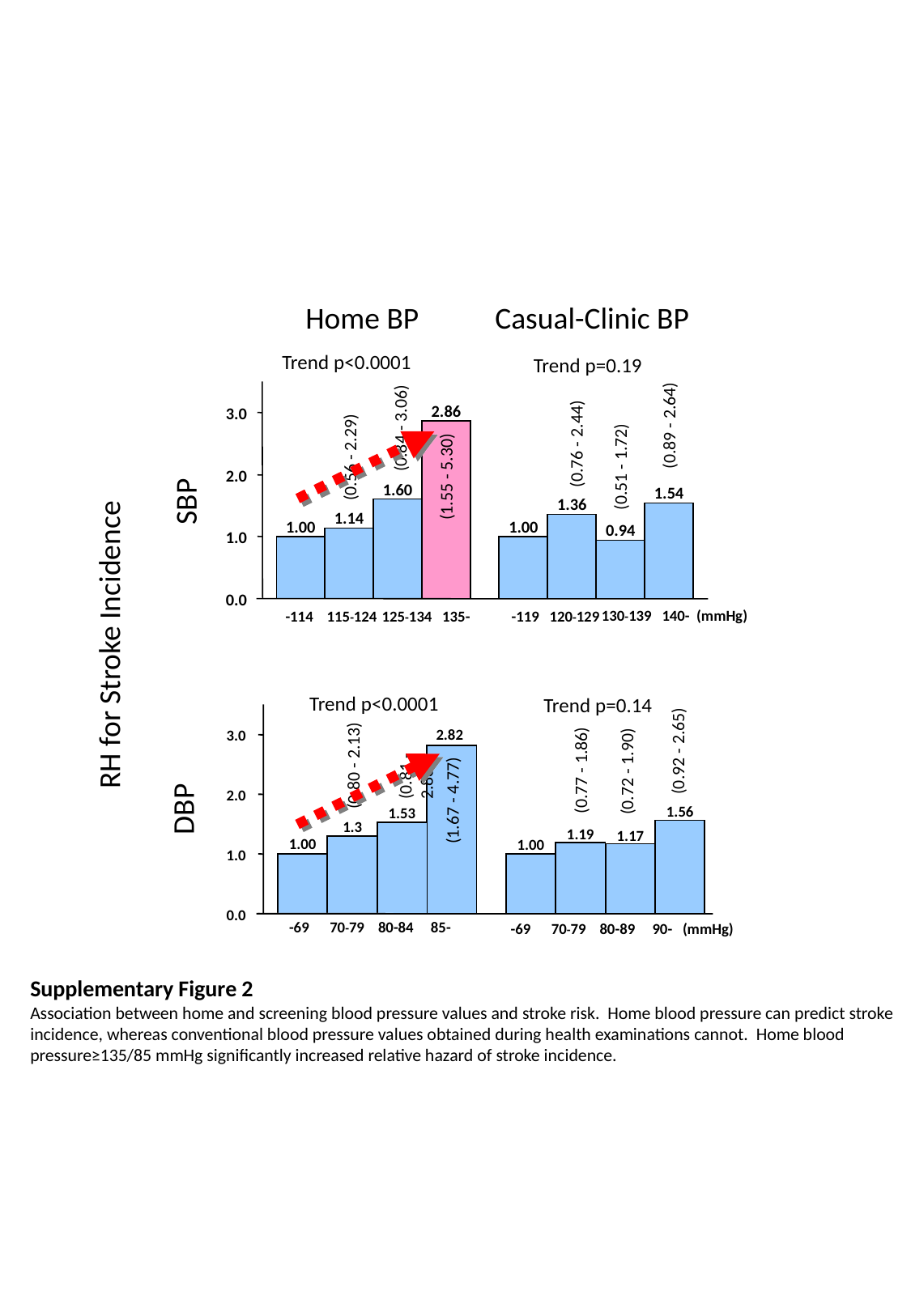

Home BP
Casual-Clinic BP
Trend p<0.0001
Trend p=0.19
(0.89 - 2.64)
(0.76 - 2.44)
(0.51 - 1.72)
1.54
1.36
1.00
0.94
2.86
(0.84 - 3.06)
(0.56 - 2.29)
(1.55 - 5.30)
1.60
1.14
1.00
3.0
2.0
SBP
1.0
0.0
130-139 140- (mmHg)
-119 120-129
-114 115-124
125-134 135-
RH for Stroke Incidence
Trend p<0.0001
Trend p=0.14
(0.92 - 2.65)
(0.77 - 1.86)
(0.72 - 1.90)
1.56
1.19
1.17
1.00
2.82
(0.81 - 2.88)
(0.80 - 2.13)
(1.67 - 4.77)
1.53
1.3
1.00
3.0
DBP
2.0
1.0
0.0
 -69 70-79 80-84 85-
 -69 70-79 80-89 90- (mmHg)
Supplementary Figure 2
Association between home and screening blood pressure values and stroke risk. Home blood pressure can predict stroke incidence, whereas conventional blood pressure values obtained during health examinations cannot. Home blood pressure≥135/85 mmHg significantly increased relative hazard of stroke incidence.

## Slide 3
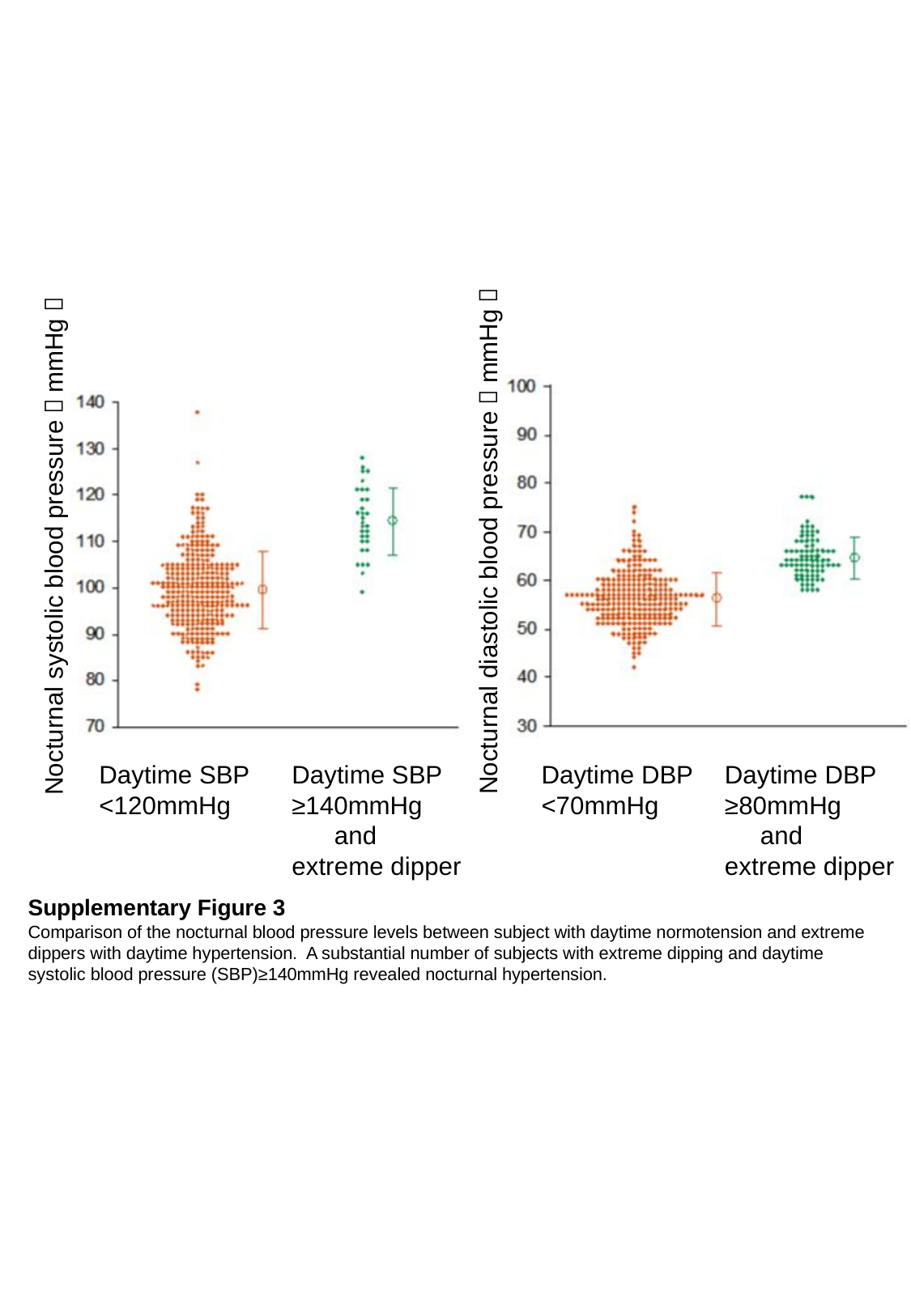

Nocturnal diastolic blood pressure（mmHg）
Nocturnal systolic blood pressure（mmHg）
Daytime SBP
≥140mmHg
 and
extreme dipper
Daytime DBP
≥80mmHg
 and
extreme dipper
Daytime SBP
<120mmHg
Daytime DBP
<70mmHg
Supplementary Figure 3
Comparison of the nocturnal blood pressure levels between subject with daytime normotension and extreme dippers with daytime hypertension. A substantial number of subjects with extreme dipping and daytime systolic blood pressure (SBP)≥140mmHg revealed nocturnal hypertension.

## Slide 4
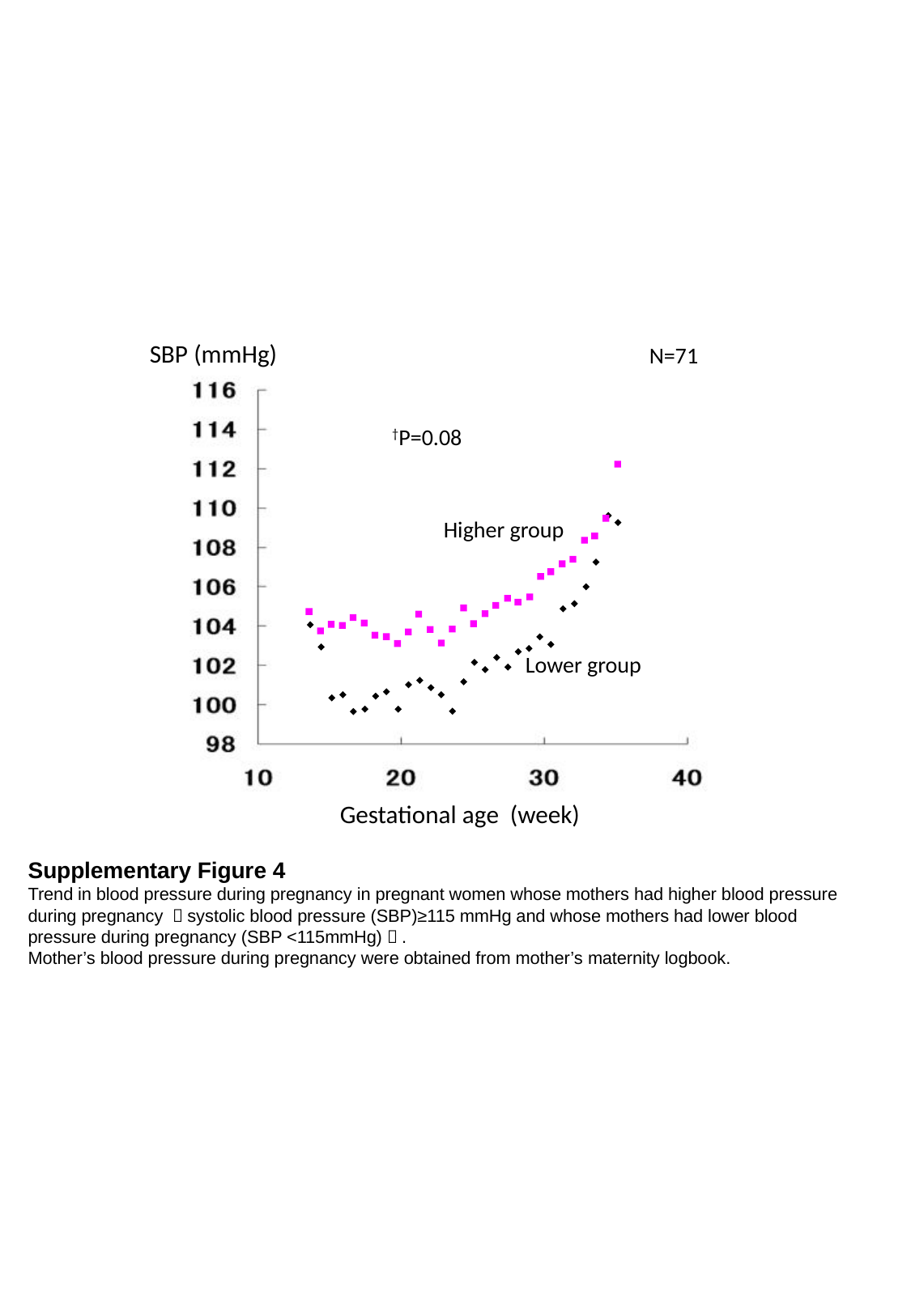

SBP (mmHg)
N=71
†P=0.08
Higher group
Lower group
Gestational age (week)
Supplementary Figure 4
Trend in blood pressure during pregnancy in pregnant women whose mothers had higher blood pressure during pregnancy ［systolic blood pressure (SBP)≥115 mmHg and whose mothers had lower blood pressure during pregnancy (SBP <115mmHg)］.
Mother’s blood pressure during pregnancy were obtained from mother’s maternity logbook.

## Slide 5
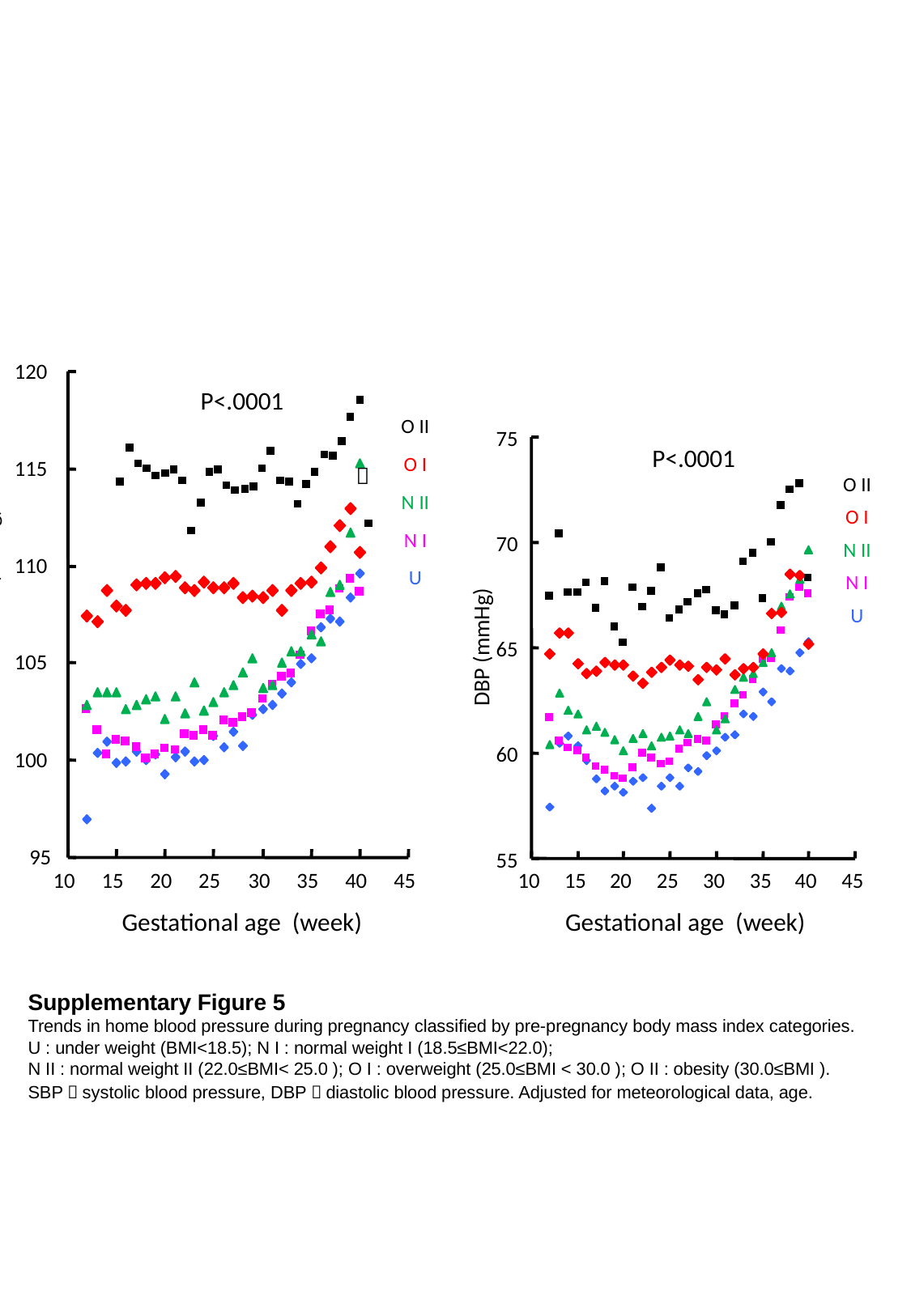

120
P<.0001
O II
75
P<.0001
O I
115
ｚ
O II
N II
O I
N I
70
N II
SBP (mmHg)
110
U
N I
U
DBP (mmHg)
65
105
60
100
95
55
10
15
20
25
30
35
40
45
35
10
15
20
25
30
40
45
Gestational age (week)
Gestational age (week)
Supplementary Figure 5
Trends in home blood pressure during pregnancy classified by pre-pregnancy body mass index categories.
U : under weight (BMI<18.5); N I : normal weight I (18.5≤BMI<22.0);
N II : normal weight II (22.0≤BMI< 25.0 ); O I : overweight (25.0≤BMI < 30.0 ); O II : obesity (30.0≤BMI ).
SBP：systolic blood pressure, DBP：diastolic blood pressure. Adjusted for meteorological data, age.

## Slide 6
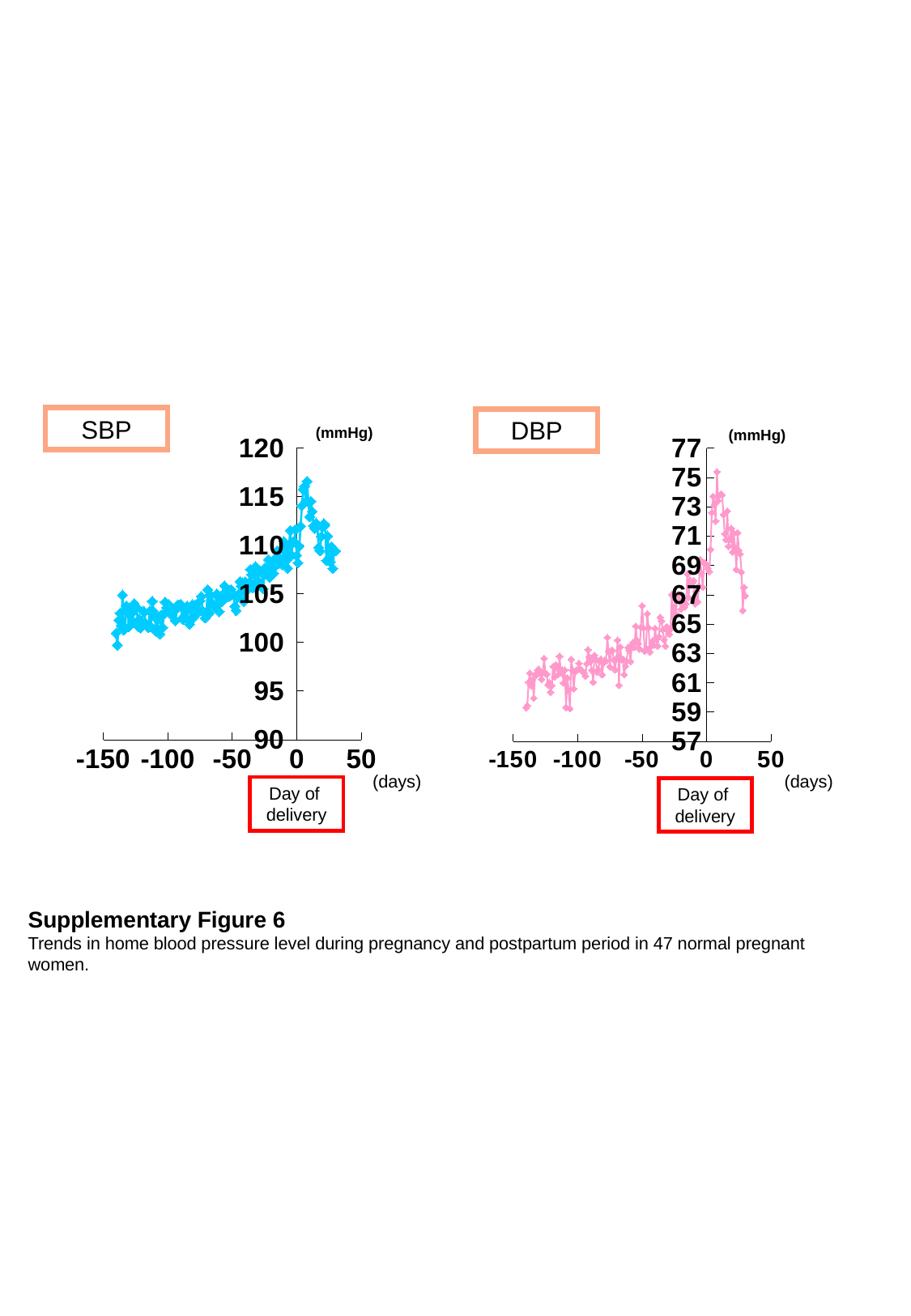

SBP
DBP
(mmHg)
(mmHg)
### Chart
| Category | 補正あり(N=54) |
|---|---|
### Chart
| Category | 補正あり(N=54) |
|---|---|Day of
delivery
Day of
delivery
(days)
(days)
Supplementary Figure 6
Trends in home blood pressure level during pregnancy and postpartum period in 47 normal pregnant women.
6
