# Supplementary material for: A personal history of research on hypertension From an encounter with hypertension to the development of hypertension practice based on out-of-clinic blood pressure measurements
Source: Hypertens Res. 2022 Sep 8;45(11):1726–42. doi: 10.1038/s41440-022-01011-1 (PMC9637554; doi:10.1038/s41440-022-01011-1)
Supplement: Supplementary file 3 — Supplementary references [file 41440_2022_1011_MOESM3_ESM.doc]

**Supplementary references 1**

***International Database on Ambulatory blood pressure monitoring in relation to Cardiovascular Outcomes (IDOCO)***

1. Kikuya M, Hansen TW, Thijs L, Bjorklund-Bodegard K, Kuznetsova T, Ohkubo T, Richart T, Torp-Pedersen C, Lind L, Ibsen H, Imai Y, Staessen JA. Diagnostic thresholds for ambulatory blood pressure monitoring based on 10-year cardiovascular risk. Circulation 2007;115:2145-2152
2. Kikuya M, Stasessen JA, Ohkubo T, Thijs L, Metoki H, Asayama K, Obara T, Inoue R, Li Y, Dolan E, Hoshi H, Hashimoto J, Totsune K, Satoh H, Wang JG, O'Brien E, Imai Y. Ambulatory arterial stiffness index and 24-hour ambulatory pulse pressure as predictors of mortality in Ohasama, Japan. Stroke 2007;38:1161-1166
3. Hansen TW, Kikuya M, Thijs L, Björklund-Bodegård K, Kuznetsova T, Ohkubo T, Richart T, Torp-Pedersen C, Lind L, Jeppesen J, Ibsen H, Imai Y, Staessen JA; IDACO Investigators. Prognostic superiority of daytime ambulatory over conventional blood pressure in four populations: a meta-analysis of 7030 individuals. J Hypertens 2007;25:1554-1564
4. Thijs L, Hansen TW, Kikuya M, Björklund-Bodegård K, Li Y, Dolan E, Tikhonoff V, Seidlerová J, Kuznetsova T, Stolarz K, Bianchi M, Richart T, Casiglia E, Malyutina S, Filipovsky J, Kawecka-Jaszcz K, Nikitin Y, Ohkubo T, Sandoya E, Wang J, Torp-Pedersen C, Lind L, Ibsen H, Imai Y, Staessen JA, O'Brien E; IDACO Investigators. The International database of ambulatory blood pressure in relation to cardiovascular outcome (IDACO): protocol and research perspectives. Blood Press Monit 2007;12:255-262
5. Boggia J, Li Y, Thijs L, Hansen TW, Kikuya M, Björklund-Bodegård K, Richart T, Ohkubo T, Kuznetsova T, Torp-Pedersen C, Lind L, Ibsen H, Imai Y, Wang J, Sandoya E, O'Brien E, Staessen JA; International Database on Ambulatory blood pressure monitoring in relation to Cardiovascular Outcomes (IDACO) investigators. Prognostic accuracy of day versus night ambulatory blood pressure: a cohort study. Lancet 2007;370:1219-1229
6. Hansen TW, Kikuya M, Thijs L, Li Y, Boggia J, Björklund-Bodegârd K, Torp-Pedersen C, Jeppesen J, Ibsen H, Staessen JA. Diagnostic thresholds for ambulatory blood pressure moving lower: a review based on a meta-analysis-clinical implications. J Clin Hypertens 2008;10:377-381
7. Li Y, Boggia J, Thijs L, Hansen TW, Kikuya M, Bjorklund-Bodegard K, Richart T, Ohkubo T, Kuznestsova T, Torp-Pedersen C, Lind L, Ibsen H, Imai Y, Wang J, Sandoya E, O'Brien E, Staessen JA, International Databese on ambulatory blood pressure monitoring in relation to cardiovascualr outcomes investigators. Is blood pressure during the night more predictive of cardiovascular outcome than during the day? Blood Press Monit 2008;13:145-147
8. Adiyaman A, Dechering DG, Boggia J, Li Y, Hansen TW, Kikuya M, Björklund-Bodegård K, Richart T, Thijs L, Torp-Pedersen C, Ohkubo T, Dolan E, Imai Y, Sandoya E, Ibsen H, Wang J, Lind L, O'Brien E, Thien T, Staessen JA, International Database on Ambulatory Blood Pressure Monitoring in Relation to Cardiovascular Outcomes Investigators. Determinants of the ambulatory arterial stiffness index in 7604 subjects from 6 populations. Hypertension 2008;52:1038-1044
9. Hansen TW, Thijs L, Li Y, Boggia J, Kikuya M, Björklund-Bodegård K, Richart T, Ohkubo T, Jeppesen J, Torp-Pedersen C, Dolan E, Kuznetsova T, Stolarz-Skrzypek K, Tikhonoff V, Malyutina S, Casiglia E, Nikitin Y, Lind L, Sandoya E, Kawecka-Jaszcz K, Imai Y, Wang J, Ibsen H, O'Brien E, Staessen JA; International Database on Ambulatory Blood Pressure in Relation to Cardiovascular Outcomes Investigators. Prognostic value of reading-to-reading blood pressure variability over 24 hours in 8938 subjects from 11 populations. Hypertension 2010;55:1049-1057
10. Li Y, Thijs L, Hansen TW, Kikuya M, Boggia J, Richart T, Metoki H, Ohkubo T, Torp-Pedersen C, Kuznetsova T, Stolarz-Skrzypek K, Tikhonoff V, Malyutina S, Casiglia E, Nikitin Y, Sandoya E, Kawecka-Jaszcz K, Ibsen H, Imai Y, Wang J, Staessen JA; International Database on Ambulatory Blood Pressure Monitoring in Relation to Cardiovascular Outcomes Investigators. Prognostic value of the morning blood pressure surge in 5645 subjects from 8 populations. Hypertension 2010;55:1040-1048
11. Stolarz-Skrzypek K, Thijs L, Richart T, Li Y, Hansen TW, Boggia J, Kuznetsova T, Kikuya M, Kawecka-Jaszcz K, Staessen JA. Blood pressure variability in relation to outcome in the International Database of Ambulatory blood pressure in relation to Cardiovascular Outcome. Hypertens Res 2010;33:757-766
12. Fan HQ, Li Y, Thijs L, Hansen TW, Boggia J, Kikuya M, Björklund-Bodegård K, Richart T, Ohkubo T, Jeppesen J, Torp-Pedersen C, Dolan E, Kuznetsova T, Stolarz-Skrzypek K, Tikhonoff V, Malyutina S, Casiglia E, Nikitin Y, Lind L, Sandoya E, Kawecka-Jaszcz K, Imai Y, Ibsen H, O'Brien E, Wang J, Staessen JA, International Database on Ambulatory Blood Pressure In Relation to Cardiovascular Outcomes Investigators. Prognostic value of isolated nocturnal hypertension on ambulatory measurement in 8711 individuals from 10 populations. J Hypertens 2010;28:2036-2045
13. Sehestedt T, Hansen TW, Li Y, Richart T, Boggia J, Kikuya M, Thijs L, Stolarz-Skrzypek K, Casiglia E, Tikhonoff V, Malyutina S, Nikitin Y, Bjorklund-Bodegard K, Kuznetsova T, Ohkubo T, Lind L, Torp-Pedersen C, Jeppesen J, Ibsen H, Imai Y, Wang J, Sandoya E, Kawecka-Jaszcz K, Staessen JA. Are blood pressure and diabetes additive or synergistic risk factors? Outcome in 8494 subjects randomly recruited from 10 populations. Hypertens Res 2011;34:714-721
14. Boggia J, Thijs L, Hansen TW, Li Y, Kikuya M, Björklund-Bodegård K, Richart T, Ohkubo T, Jeppesen J, Torp-Pedersen C, Dolan E, Kuznetsova T, Olszanecka A, Tikhonoff V, Malyutina S, Casiglia E, Nikitin Y, Lind L, Maestre G, Sandoya E, Kawecka-Jaszcz K, Imai Y, Wang J, Ibsen H, O'Brien E, Staessen JA, International Database on Ambulatory blood pressure in relation to Cardiovascular Outcomes Investigators. Ambulatory blood pressure monitoring in 9357 subjects from 11 populations highlights missed opportunities for cardiovascular prevention in women. Hypertension 2011;57:397-405
15. Franklin SS, Thijs L, Hansen TW, Li Y, Boggia J, Kikuya M, Björklund-Bodegård K, Ohkubo T, Jeppesen J, Torp-Pedersen C, Dolan E, Kuznetsova T, Stolarz-Skrzypek K, Tikhonoff V, Malyutina S, Casiglia E, Nikitin Y, Lind L, Sandoya E, Kawecka-Jaszcz K, Imai Y, Wang J, Ibsen H, O'Brien E, Staessen JA, International Database on Ambulatory Blood Pressure in Relation to Cardiovascular Outcomes Investigators. Significance of white-coat hypertension in older persons with isolated systolic hypertension: a meta-analysis using the International Database on Ambulatory Blood Pressure Monitoring in Relation to Cardiovascular Outcomes population. Hypertension 2012;59:564-571
16. Franklin SS, Thijs L, Li Y, Hansen TW, Boggia J, Liu Y, Asayama K, Björklund-Bodegård K, Ohkubo T, Jeppesen J, Torp-Pedersen C, Dolan E, Kuznetsova T, Stolarz-Skrzypek K, Tikhonoff V, Malyutina S, Casiglia E, Nikitin Y, Lind L, Sandoya E, Kawecka-Jaszcz K, Filipovský J, Imai Y, Wang J, Ibsen H, O'Brien E, Staessen JA; International Database on Ambulatory blood pressure in relation to Cardiovascular Outcomes (IDACO) Investigators. Response to Masked hypertension in untreated and treated patients with diabetes mellitus: attractive but questionable interpretations and response to Is masked hypertension related to diabetes mellitus? Hypertension 2013;62:e23-25
17. Schutte R, Thijs L, Asayama K, Boggia J, Li Y, Hansen TW, Liu YP, Kikuya M, Björklund-Bodegård K, Ohkubo T, Jeppesen J, Torp-Pedersen C, Dolan E, Kuznetsova T, Stolarz-Skrzypek K, Tikhonoff V, Malyutina S, Casiglia E, Nikitin Y, Lind L, Sandoya E, Kawecka-Jaszcz K, Filipovsky J, Imai Y, Wang J, Ibsen H, O'Brien E, Staessen JA, International Database on Ambulatory blood pressure in relation to Cardiovascular Outcomes (IDACO) Investigators. Double product reflects the predictive power of systolic pressure in the general population: evidence from 9,937 participants. Am J Hypertens 2013;26:665-672
18. Boggia J, Thijs L, Li Y, Hansen TW, Kikuya M, Bjorklund-Bodegard K, Ohkubo T, Jeppesen J, Torp-Pedersen C, Dolan E, Kuznetsova T, Stolarz-Skrzypek K, Tikhonoff V, Malyutina S, Casiglia E, Nikitin Y, Lind L, Schwedt E, Sandoya E, Kawecka-Jaszcz K, Filipovsky J, Imai Y, Wang J, Ibsen H, O'Brien E, Staessen JA, International Database on Ambulatory blood pressure in relation to Cardiovascular Outcomes (IDACO) Investigators. Risk stratification by 24-hour ambulatory blood pressure and estimated glomerular filtration rate in 5322 subjects from 11 populations. Hypertension 2013;61:18-26
19. Franklin SS, Thijs L, Li Y, Hansen TW, Boggia J, Liu Y, Asayama K, Bjorklund-Bodegard K, Ohkubo T, Jeppesen J, Torp-Pedersen C, Dolan E, Kuznetsova T, Stolarz-Skrzypek K, Tikhonoff V, Malyutina S, Casiglia E, Nikitin Y, Lind L, Sandoya E, Kawecka-Jaszcz K, Filipovsky J, Imai Y, Wang J, Ibsen H, O'Brien E, Staessen JA, International Database on Ambulatory blood pressure in relation to Cardiovascular Outcomes (IDACO) Investigators. Masked ｈypertension in ｄiabetes ｍellitus: ｔreatment mplications for clinical practice. Hypertension 2013;61:964-971
20. Gu YM, Thijs L, Li Y, Asayama K, Boggia J, Hansen TW, Liu YP, Ohkubo T, Björklund-Bodegård K, Jeppesen J, Dolan E, Torp-Pedersen C, Kuznetsova T, Stolarz-Skrzypek K, Tikhonoff V, Malyutina S, Casiglia E, Nikitin Y, Lind L, Sandoya E, Kawecka-Jaszcz K, Imai Y, Mena LJ, Wang J, O'Brien E, Verhamme P, Filipovský J, Maestre GE, Staessen JA, International Database on Ambulatory blood pressure in relation to Cardiovascular Outcomes (IDACO) Investigators. Outcome-driven thresholds for ambulatory pulse pressure in 9938 participants recruited from 11 populations. Hypertension 2014;63:229-237
21. Mena LJ, Maestre GE, Hansen TW, Thijs L, Liu Y, Boggia J, Li Y, Kikuya M, Björklund-Bodegård K, Ohkubo T, Jeppesen J, Torp-Pedersen C, Dolan E、Kuznetsova T, Stolarz-Skrzypek K, Tikhonoff V, Malyutina S, Casiglia E, Nikitin Y, Lind L, Sandoya E, Kawecka-Jaszcz K, Filipovskŷ J, Imai Y, Wang J, O'Brien E, Staessen JA ; International Database on Ambulatory Blood Pressure in Relation to Cardiovascular Outcomes (IDACO) Investigators. How many measurements are needed to estimate blood pressure variability without loss of prognostic information? Am J Hypertens 2014;27:46-55
22. Li Y, Thijs L, Boggia J, Asayama K, Hansen TW, Kikuya M, Björklund-Bodegård K, Ohkubo T, Jeppesen J, Torp-Pedersen C, Dolan E, Kuznetsova T, Stolarz-Skrzypek K, Tikhonoff V, Malyutina S, Casiglia E, Nikitin Y, Lind L, Sandoya E, Kawecka-Jaszcz K, Filipovsky J, Imai Y, Ibsen H, O'Brien E, Wang J, Staessen JA, International Database on Ambulatory blood pressure in relation to Cardiovascular Outcomes (IDACO) Investigators. Blood pressure load does not add to ambulatory blood pressure level for cardivascular risk stratification. Hypertension 2014;63:925-933
23. Hansen TW, Thijs L, Li Y, Boggia J, Liu Y, Asayama K, Kikuya M, Björklund-Bodegård K, Ohkubo T, Jeppesen J, Torp-Pedersen C, Dolan E, Kuznetsova T, Stolarz-Skrzypek K, Tikhonoff V, Malyutina S, Casiglia E, Nikitin Y, Lind L, Sandoya E, Kawecka-Jaszcz K, Filipovský J, Imai Y, Wang J, O7brien E, Staessen JA. Ambulatory blood pressure monitoring for risk stratification in obese and non-obese subjects from 10 popularions. J Hum Hypertens 2014;28:535-542
24. Brguljan-Hitij J, Thijs L, Li Y, Hansen TW, Boggia J, Liu YP, Asayama K, Wei FF, Bjoklund-Bodegard K, Gu YM, Ohkubo T, Jeppesen J, Torp-Pedersen C, Dolan E, Kuznetsova T, Katarzyna SS, Tikhonoff V, Malyutina S, Casiglia E, Nikitin Y, Lind L, Sandoya E, Kawecka-Jaszcz K, Filipovsky J, Imai Y, Wang J, O'Brien E, Staessen JA, International Database on Ambulatory blood pressure in relation to Cardiovascular Outcome Investigators. Risk stratification by ambulatory blood pressure monitoring across JNC classes of conventional blood pressure. Am J Hypertens 2014;27:956-965
25. Asayama K, Thijs L, Li Y, Gu YM, Hara A, Liu YP, Zhang Z, Wei FF, Lujambio I, Mena LJ, Boggia J, Hansen TW, Björklund-Bodegård K, Nomura K, Ohkubo T, Jeppesen J, Torp-Pedersen C, Dolan E, Stolarz-Skrzypek K, Malyutina S, Casiglia E, Nikitin Y, Lind L, Luzardo L, Kawecka-Jaszcz K, Sandoya E, Filipovský J, Maestre GE, Wang J, Imai Y, Franklin SS, O'Brien E, Staessen JA, International Database on Ambulatory Blood Pressure in Relation to Cardiovascular Outcomes (IDACO) Investigators. Setting thresholds to varying blood pressure monitoring intervals differentially affects risk estimates associated with white-coat and masked hypertension in the population. Hypertension 2014;64:935-942
26. Conen D, Aeschbacher S, Thijs L, Li Y, Boggia J, Asayama K, Hansen TW, Kikuya M, Björklund-Bodegård K, Ohkubo T, Jeppesen J, Gu YM, Torp-Pedersen C, Dolan E, Kuznetsova T, Stolarz-Skrzypek K, Tikhonoff V, Schoen T, Malyutina S, Casiglia E, Nikitin Y, Lind L, Sandoya E, Kawecka-Jaszcz K, Mena L, Maestre GE, Filipovský J, Imai Y, O'Brien E, Wang JG, Risch L, Staessen JA. Age-specific differences between conventional and ambulatory daytime blood pressure values. Hypertension 2014;64:1073-1079
27. Li Y, Wei FF, Thijs L, Boggia J, Asayama K, Hansen TW, Kikuya M, Björklund-Bodegard K, Ohkubo T, Jeppesen JL, Gu YM, Torp-Pedersen C, Dolan E, Liu YP, Kuznetsova T, Stolarz-Skrzypek K, Tikhonoff V, Malyutina S, Casiglia E, Nikitin Y, Lind L, Sandoya E, Kawecka-Jaszcz K, Mena L, Maestre GE, Filipovský J, Imai Y, O'Brien E, Wang JG, Staessen JA. Ambulatory hypertension Subtypes and 24-hour Systolic and Diastolic Blood Pressure as distinct outcome predictors in 8341 untreated people recruited from 12 populations. Circulation 2014;130:466-474
28. Franklin SS, Thijs L, Asayama K, Li Y, Hansen TW, Boggia J, Jacobs L, Zhang Z, Kikuya M, Björklund-Bodegård K, Ohkubo T, Yang WY, Jeppesen J, Dolan E, Kuznetsova T, Stolarz-Skrzypek K, Tikhonoff V, Malyutina S, Casiglia E, Nikitin Y, Lind L, Sandoya E, Kawecka-Jaszcz K, Filipovský J, Imai Y, Wang JG, O'Brien E, Staessen JA; IDACO Investigators. The cardiovascular risk of white-coat ypertension. J Am Coll Cardiol 2016;68:2033-2043
29. Yang WY, Thijs L, Zhang ZY, Asayama K, Boggia J, Hansen TW, Ohkubo T, Jeppesen J, Stolarz-Skrzypek K, Malyutina S, Casiglia E, Nikitin Y, Li Y, Wang JG, Imai Y, Kawecka-Jaszcz K, O'Brien E, Staessen JA, International Database on Ambulatory blood pressure in relation to Cardiovascular Outcomes (IDACO) Investigators. Evidence-based proposal for the number of ambulatory readings required for assessing blood pressure level in research settings: an analysis of the IDACO database. Blood Press 2018;27:341-350
30. Yang WY, Melgarejo JD, Thijs L, Zhang ZY, Boggia J, Wei FF, Hansen TW, Asayama K, Ohkubo T, Jeppesen J, Dolan E, Stolarz-Skrzypek K, Malyutina S, Casiglia E, Lind L, Filipovský J, Maestre GE, Li Y, Wang JG, Imai Y, Kawecka-Jaszcz K, Sandoya E, Narkiewicz K, O'Brien E, Verhamme P, Staessen JA; International Database on Ambulatory Blood Pressure in Relation to Cardiovascular Outcomes (IDACO) Investigators. Association of office and ambulatory blood pressure with mortality and cardiovascular outcomes. JAMA 2019;322:409-420
31. Cheng YB, Thijs L, Zhang ZY, Kikuya M, Yang WY, Melgarejo JD, Boggia J, Wei FF, Hansen TW, Yu CG, Asayama K, Ohkubo T, Dolan E, Stolarz-Skrzypek K, Malyutina S, Casiglia E, Lind L, Filipovský J, Maestre GE, Imai Y, Kawecka-Jaszcz K, Sandoya E, Narkiewicz K, Li Y, O'Brien E, Wang JG, Staessen JA. Outcome-sriven thresholds for ambulatory blood pressure based on the new American College of Cardiology/American Heart Association classification of hypertension. Hypertension 2019;74:776-783
32. Li Y, Thijs L, Zhang ZY, Asayama K, Hansen TW, Boggia J, Björklund-Bodegård K, Yang WY, Niiranen TJ, Ntineri A, Wei FF, Kikuya M, Ohkubo T, Dolan E, Hozawa A, Tsuji I, Stolarz-Skrzypek K, Huang QF, Melgarejo JD, Tikhonoff V, Malyutina S, Casiglia E, Nikitin Y, Lind L, Sandoya E, Aparicio L, Barochiner J, Gilis-Malinowska N, Narkiewicz K, Kawecka-Jaszcz K, Maestre GE, Jula AM, Johansson JK, Kuznetsova T, Filipovský J, Stergiou G, Wang JG, Imai Y, O'Brien E, Staessen JA, International Database on Ambulatory and Home Blood Pressure in Relation to Cardiovascular Outcome Investigators. Opposing age-related trends in absolute and relative risk of dverse ealth outcomes associated with out-of-office blood pressure. Hypertension 2019;74:1333-1342
33. Melgarejo JD, Yang WY, Thijs L, Li Y, Asayama K, Hansen TW, Wei FF, Kikuya M, Ohkubo T, Dolan E, Stolarz-Skrzypek K, Huang QF, Tikhonoff V, Malyutina S, Casiglia E, Lind L, Sandoya E, Filipovský J, Gilis-Malinowska N, Narkiewicz K, Kawecka-Jaszcz K, Boggia J, Wang JG, Imai Y, Vanassche T, Verhamme P, Janssens S, O'Brien E, Maestre GE, Staessen JA, Zhang ZY, International Database on Ambulatory Blood Pressure in Relation to Cardiovascular Outcome Investigators. Association of fatal and nonfatal cardiovascular outcomes with 24-hour mean arterial pressure. Hypertension 2021;77:39-48

**Supplementary ferences 2**

***International Database of HOme blood pressure in relation to Cardiovascular Outcomes (IDHOCO)***

1. Niiranen TJ, Thijs L, Asayama K, Johansson JK, Ohkubo T, Kikuya M, Boggia J, Hozawa A, Sandoya E, Stergiou GS, Tsuji I, Jula AM, Imai Y, Staessen JA; IDHOCO Investigators. The International Database of HOme blood pressure in relation to Cardiovascular Outcome (IDHOCO): moving from baseline characteristics to research perspectives. Hypertens Res 2012;35:1072-1079
2. Niiranen TJ, Asayama K, Thijs L, Johansson JK, Ohkubo T, Kikuya M, Boggia J, Hozawa A, Sandoya E, Stergiou GS, Tsuji I, Jula AM, Imai Y, Staessen JA、 International Database of Home blood pressure in relation to Cardiovascular Outcome Investigators. Outcome-driven thresholds for home blood pressure measurement: international database of home blood pressure in relation to cardiovascular outcome. Hypertension 2013;61:27-34
3. Stergiou GS, Asayama K, Thijs L, Kollias A, Niiranen TJ, Hozawa A, Boggia J, Johansson JK, Ohkubo T, Tsuji I, Jula AM, Imai Y, Staessen JA、 International Database on Home blood pressure in relation to Cardiovascular Outcome (IDHOCO) Investigators. Prognosis of white-coat and masked hypertension: international database of home blood pressure in relation to cardiovascular outcome. Hypertension 2014;63:675-682
4. Asayama K, Thijs L, Brguljan-Hitij J, Niiranen TJ, Hozawa A, Boggia J, Aparicio LS, Hara A, Johansson JK, Ohkubo T, Tzourio C, Stergiou GS, Sandoya E, Tsuji I, Jula AM, Imai Y, Staessen JA; International Database of Home Blood Pressure in Relation to Cardiovascular Outcome (IDHOCO) investigators. Risk stratification by self-measured home blood pressure across categories of conventional blood pressure: A participant-level meta-analysis. PLOS Medicine 2014;11:e1001591
5. Aparicio LS, Thijs L, Asayama K, Barochiner J, Boggia J, Gu YM, Cuffaro PE, Liu YP, Niiranen TJ, Ohkubo T, Johansson JK, Kikuya M, Hozawa A, Tsuji I, Imai Y, Sandoya E, Stergiou GS, Waisman GD, Staessen JA、 International Database on Home blood pressure in relation to Cardiovascular Outcome (IDHOCO) Investigators. Reference frame for home pulse pressure based on cardiovascular risk in 6470 subjects from 5 populations. Hypertens Res 2014;37:672-678
6. Nomura K, Asayama K, Thijs L, Niiranen TJ, Lujambio I, Boggia J, Hozawa A, Ohkubo T, Hara A, Johansson JK, Sandoya E, Kollias A, Stergiou GS, Tsuji I, Jula AM, Imai Y, Staessen JA. Thresholds for conventional and home blood pressure by sex and age in 5018 participants from 5 populations. Hypertension 2014;64:695-701
7. Niiranen TJ, Asayama K, Thijs L, Johansson JK, Hara A, Hozawa，A, Tsuji I, Ohkubo T, Jula AM, Imai Y, Staessen JA, IDHOCO Investigators. Optimal Number of Days for Home Blood Pressure Measurement. Am J Hypertens 2015;28:595-603
8. Aparicio LS, Thijs L, Boggia J, Jacobs L, Barochiner J, Odili AN, Alfie J, Asayama K, Cuffaro PE, Nomura K, Ohkubo T, Tsuji I, Stergiou GS, Kikuya M, Imai Y, Waisman GD, Staessen JA; International Database on Home Blood Pressure in Relation to Cardiovascular Outcome (IDHOCO) Investigators. Defining thresholds for home blood pressure monitoring in octogenarians. Hypertension 2015;66:865-873
9. Odili AN, Thijs L, Hara A, Wei FF, Ogedengbe JO, Nwegbu MM, Aparicio LS, Asayama K, Niiranen TJ, Boggia J, Luzardo L, Jacobs L, Stergiou GS, Johansson JK, Ohkubo T, Jula AM, Imai Y, O'Brien E, Staessen JA. Prevalence and ｄeterminants of masked hypertension among black Nigerians compared with a reference population. Hypertension 2016;67:1249-1255
10. Ntineri A, Stergiou GS, Thijs L, Asayama K, Boggia J, Boubouchairopoulou N, Hozawa A, Imai Y, Johansson JK, Jula AM, Kollias A, Luzardo L, Niiranen TJ, Nomura K, Ohkubo T, Tsuji I, Tzourio C, Wei FF, Staessen JA. Relationship between office and home blood pressure with increasing age: The International Database of HOme blood pressure in relation to Cardiovascular Outcome (IDHOCO). Hypertens Res 2016;39:612-617
11. Juhanoja EP, Niiranen TJ, Johansson JK, Puukka PJ, Thijs L, Asayama K, Langén VL, Hozawa A, Aparicio LS, Ohkubo T, Tsuji I, Imai Y, Stergiou GS, Jula AM, Staessen JA, International Database on HOme blood pressure in relation to Cardiovascular Outcome(IDHOCO) Investigators. Outcome-driven thresholds for increased home blood pressure variability. Hypertension 2017;69:599-607

**Supplementary references 3**

***Asia Pacific Cohort Studies Collaboration***

1. Lawes CM, Bennett DA, Parag V, Woodward M, Whitlock G, Lam TH, Suh I, Rodgers A ;Asia Pacific Cohort Studies Collaboration. Blood pressure indices and cardiovascular disease in the Asia. Pacific region: a pooled analysis. Hypertension 2003;42:69-75
2. Lawes CM, Rodgers A, Bennett DA, Parag V, Suh I, Ueshima H, MacMahon S; Asia Pacific Cohort Studies Collaboration. Blood pressure and cardiovascular disease in the Asia Pacific region. J Hypertens 2003;21:707-716
3. Woodward M, Zhang X, Barzi F, Pan W, Ueshima H, Rodgers A, MacMahon S; Asia Pacific Cohort Studies Collaboration. The effects of diabetes on the risks of major cardiovascular diseases and death in the Asiapacific region. Diabetes Care 2003;26:360-366
4. Patel A, Barzi F, Jamrozik K, Lam TH, Ueshima H, Whitlock G, Woodward M; Asia Pacific Cohort Studies Collaboration. Serum triglycerides as a risk factor for cardiovascular diseases in the Asia-Pacific Region. Circulation 2004;110:2678-2686
5. Asia Pacific Cohort Studies Collaboration. Joint effects of systolic blood pressure and serum cholesterol on cardiovascular disease in the Asia Pacific region. Circulation 2005;112:3384-3390
6. Feigin V, Parag V, Lawes CM, Rodgers A, Suh I, Woodward M, Jamrozik K, Ueshima H; Asia Pacific Cohort Studies Collaboration. Smoking and elevated blood pressure are the most important risk factors for subarachnoid hemorrhage in the Asia-Pacific region: an overview of 26 cohorts involving 306,620 participants. Stroke 2005;36:1360-1365
7. Asia Pacific Cohort Studies Collaboration； Patel A, Barzi F, Woodard M, Ni Mhurchu C, Ohkubo T, Lam TH, Welborn T. An evaluation of metabolic risks for coronary death in the Asia Pacific region. Diabetes Res Clin Pract 2006;74:274-281
8. Woodward M, Barzi F, Martiniuk A, Fang X, Gu DF, Imai Y, Lam TH, Pan WH, Roders M, Suh T, Jee SH, Ueshima H, Huxley R. Cohort profile: the Asia pacific cohort studies collaboration. Int J Epidemiol 2006;35:1412-1416
9. Ansary-Moghaddam A, Huxley R, Barzi F, Lawes C, Ohkubo T, Fang X, Jee SH, Woodward M; Asia Pacific Cohort Studies Collaboration. The effect of modifiable risk factors on pancreatic cancer mortality in populations of the Asia-Pacific region. Cancer Epidemiol Biomarkers Prev 2006;15:2435-2440
10. Asia Pacific Cohort Studies Collaboration； Ni Mhurchu C, Parag V, Nakamura M, Patel A, Rodgers A, Lam TH. Body mass index and risk of diabetes mellitus in the Asia-Pacific region. Asia Pac J Clin Nutr 2006;15:127-133
11. Asia Pacific Cohort Studies Collaboration. Coronary risk prediction for those with and without diabetes. Eur J Cardiovasc Prev Rehabil 2006;13:30-36
12. Asia Pacific Cohort Studies Collaboration. Central obesity and risk of cardiovascular disease in the Asia Pacific Region. Asia Pac J Clin Nutr 2006;15:287-292
13. Asia Pacific Cohort Studies Collaboration, Huxley R, Ansary-Mohaddam A, Huxley R, Barzi F, Lam TH, Jamrozik K, Ohkubo T, Fang X, Sun HJ, Woodward M, Gu DF, Rodgers A, Imai Y, Pan WH, Suh I, Sun HJ, Ueshima H. The impact of modifiable risk factors on mortality from prostate cancer in populations of the Asia-Pacific region. Asian Pacific J Cancer Prev 2007;8:199-205
14. Woodward M, Barzi F, Feigin V, Gu D, Huxley R, Nakamura K, Patel A, Ho S, Jamrozik K; Asia Pacific Cohort Studies Collaboration. Associations between high-density lipoprotein cholesterol and both stroke and coronary heart disease in the Asia Pacific region. Eur Heart J 2007;28:2653-2660
15. Asia Pacific Cohort Studies Collaboration；Barzi F, Patel A, Gu D, Sritara T, Lam TH, Rodgers A, Woodward M. Cholesterol, diabetes and major cardiovascular diseases in the Asia-Pacific region. Diabetologia 2007;50:2289-2297
16. Asia Pacific Cohort Studies Collaboration. Cardiovascular risk prediction tools for populations in Asia. J Epidemiol Community Health 2007;61:115-121
17. Martiniuk ALC, Lee CMY, Lawes CMM, Ueshima H, Suh II, Lam TH, Gu D, Feigin V, Jamrozik K, Ohkubo T, Woodward M; Asia-Pacific Cohort Studies Collaboration. Hypertension: its prevalence and population-attributable fraction for mortality from cardiovascular disease in the Asia-Pacific region. J Hypertens 2007;25:73-79
18. Asia Pacific Cohort Studies Collaboration. The burden of overweight and obesity in the Asia-Pacific region. Obes Rev 2007;8:191-196
19. Huxley R, Jamrozik K, Ham TH, Barzi F, Ansary-Moghannam A, Jiang CQ, Sun L, Woodwar M on behalf of the Asia Pacific cohort studies collaboration. Impact of smoking and smoking cessation on lung cancer mortality in the Asia-Pacific Region. Am J Epidemiol 2007;165:1280-1286
20. Lee CMY, Huxley RR, Lam TH, Martiniuk AL, Ueshema H, Pan WH, Welborn T, Woodward M; Asia Pacific Cohort Studies Collaboration. Prevalence of diabetes mellitus and population attributable fractions for coronary heart disease and stroke mortality in the WHO South-East Asia and Western Pacific regions. Asia Pac J Clin Nutr 2007;16:187-192
21. Lee CMY, Barzi F, Woodward M, Batty GD, Giles GG, Wong JW, Jamrozik K, Lam TH, Ueshima H, Kim HC, Gu DF, Schooling M, Huxley RR ; for The Asia Pacific Cohort Studies Collaboration. Adult height and the risks of cardiovascular disease and major causes of death in the Asia-Pacific region: 21,000 deaths in 510,000 men and women. Int J Epidemiol 2009;38:1060-1071
22. Ansary-Moghaddam A, Martiniuk A, Lam TH, Jamrozik K, Tamakoshi A, Fang X, Suh Il, Barzi F, Huxley R and Woodward M: the Asia Pacific Cohort Studies Collaboration. Smoking and the Risk of Upper Aero Digestive Tract Cancers for Men and Women in the Asia-Pacific Region. Int J Environ Res Public Health 2009;6:1358-1370
23. Nakamura K, Barzi F, Huxley R, Lam T-H, Suh I, Woo J, Kim C H, Feigin L V, Gu D, Woodward M, Asia Pacific Cohort Studies Collaboration. Does cigarette smoking exacerbate the effect of total cholesterol and high-density lipoprotein cholesterol on the risk of cardiovascular diseases? Heart 2009;95;909-916
24. Kengne AP, Nakamura K, Barzi F, Lam TH, Huxley R, Gu D, Patel A, Kim HC, Woodward M; Asia Pacific Cohort Study Collaboration. Smoking, diabetes and cardiovascular diseases in men in the Asia Pacific region. J Diabetes 2009;1:173-181
25. Batty GD, Barzi F, Huxley R, Chang CY, Jee SH, Jamrozik K, Kim HC, Fang X, Lam TH, Woodward M; APCSC Writing Committee. Obesity and liver cancer mortality in Asia: The Asia Pacific Cohort Studies Collaboration. Cancer Epidemiol 2009;33:469-472
26. O'Seaghdha CM, Perkovic V, Lam TH, McGinn S, Barzi F, Gu DF, Cass A, Suh I, Muntner P, Giles G G., Ueshima H, Woodward M, Huxley R and on behalf of the Asia Pacific Cohort Studies Collaboration. Blood pressure is a major risk factor for renal death: an analysis of 560 352 participants from the Asia-Pacific region. Hypertension 2009;54:509-515
27. Parr CL, Batty GD, Lam TH, Barzi F, Fang X, Ho SC, Jee SH, Ansary-Moghaddam A, Jamrozik K, Ueshima H, Woodward M, Huxley RR; Asia-Pacific Cohort Studies Collaboration. Body-mass index and cancer mortality in the Asia-Pacific Cohort Studies Collaboration: pooled analyses of 424519 participans. Lancet Oncol 2010;11:741-752
28. Lam EKK, Batty GD, Huxley RR, Martiniuk ALC, Barzi F, Lam TH, Lawes CMM, Giles GG, Welborn T, Ueshima H, Tamakoshi A, Woo J, Kim HC, Fang X, Czernichow S, Woodward M; Asia Pacific Cohort Studies Collaboration. Associations of diabetes mellitus with site-specific cancer mortality in the Asia-Pacific region. Ann Oncol 2011;22:730-738
29. Woodward M, Tsukinoki-Murakami R, Murakami Y, Suh I, Fang X, Ueshima H, Lam TH; Asia-Pacific Cohort Studies Collaboration. The epidemiology of stroke amongst women in the Asia-Pacific region. Womens Health (Lond Engl) 2011;7:305-317
30. Arima H, Murakami Y, Lam TH, Kim HC, Ueshima H, Woo J, Suh I, Fang X, Woodward M; Asia Pacific Cohort Studies Collaboration. Effects of prehypertension and hypertension subtype on cardiovascular disease in the Asia-Pacific Region. Hypertension 2012;59:1118-1123
31. Murakami Y, Huxley RR, Lam T-H, Tsukinoki R, Fang X, Kim HC, Woodward M; Asia Pacific Cohort Studies Collaboration. Diabetes, body mass index and the excess risk of coronary heart disease, ischemic and hemorrhagic stroke in the Asia Pacific Cohort Studies Collaboration. Prev Med 2012;54:38-41

**Supplementary references 4**

***Blood Pressure Lowering Treatment Trialists' Collaboration (BPLTTC)***

1. Turnbull F, Neal B, Algert C, Chalmers J, Chapman N, Cutler J, Woodward M, MacMahon S; Blood Pressure Lowering Treatment Trialists' Collaboration. Effects of different blood pressure-lowering regimens on major cardiovascular events in individuals with and without diabetes mellitus: results of prospectively designed overviews of randomized trials. Arch Intern Med 2005;165:1410-1419
2. Blood Pressure Lowering Treatment Trialists' Collaboration； Turnbull F, Neal B, Pfeffer M, Kostis J, Algert C, Woodward M, Chalmers J, Zanchetti A, MacMahon S. Blood pressure-dependent and independent effects of agents that inhibit the renin-angiotensin system. J Hypertens 2007;25:951-958
3. Blood Pressure Lowering Treatment Trialists' Collaboration； Turnbull F, Neal B, Ninomiya T, Algert C, Arima H, Barzi F, Bulpitt C, Chalmers J, Fagard R, Gleason A, Heritier S, Li N, Perkovic V, Woodward M, MacMahon S. Effects of different regimens to lower blood pressure on major cardiovascular events in older and younger adults: meta-analysis of randomised trials. BMJ 2008;336:1121-1123
4. Czernichow S, Zanchetti A, Turnbull F, Barzi F, Ninomiya T, Kengne AP, Lambers Heerspink HJ, Perkovic V, Huxley R, Arima H, Patel A, Chalmers J, Woodward M, MacMahon S, Neal B, Blood Pressure Lowering Treatment Trialists' Collaboration. The effects of blood pressure reduction and of different blood pressure-lowering regimens on major cardiovascular events according to baseline blood pressure: meta-analysis of randomized trials. J Hypertens 2011;29:4-16
5. Blood Pressure Lowering Treatment Trialists' Collaboration. Blood pressure-lowering treatment based on cardiovascular risk: a meta-analysis of individual patient data. Lancet 2014;384:591-598
6. Rahimi K, Canoy D, Nazarzadeh M, Salimi-Khorshidi G, Woodward M, Teo K, Davis BR, Chalmers J, Pepine CJ; Blood Pressure Lowering Treatment Trialists’ Collaboration. Investigating the stratified efficacy and safety of pharmacological blood pressure-lowering: an overall protocol for individual patient-level data meta-analyses of over 300 000 randomised participants in the new phase of the Blood Pressure Lowering Treatment Trialists' Collaboration (BPLTTC). BMJ Open 2019;9:e028698
7. Blood Pressure Lowering Treatment Trialists' Collaboration. Pharmacological blood pressure lowering for primary and secondary prevention of cardiovascular disease across different levels of blood pressure: an individual participant-level data meta-analysis. Lancet 2021;397:1625-1636
8. Blood Pressure Lowering Treatment Trialists' Collaboration. Age-stratified and blood-pressure-stratified effects of blood-pressure-lowering pharmacotherapy for the prevention of cardiovascular disease and death: an individual participant-level data meta-analysis. Lancet 2021;398:1053-1064

**Supplementaｒｙ references 5**

***Ambulatory Blood Pressure (ABP) International study***

1. Cahalin LP, Forman DE, Chase P, Guazzi M, Myers J, Bensimhon D, Peberdy MA, Ashley E, West E, Arena R (Ohasama study). The prognostic significance of heart rate recovery is not dependent upon maximal effort in patients with heart failure. Int J Cardiol 2013;168:1496-1501
2. Palatini P, Reboldi G, Beilin LJ, Casiglia E, Eguchi K, Imai Y, Kario K, Ohkubo T, Pierdomenico SD, Schwartz JE, Wing L, Verdecchia P. Added predictive value of night-time blood pressure variability for cardiovascular events and mortality: the Ambulatory Blood Pressure-International Study. Hypertension 2014;64:487-493
3. Palatini P, Reboldi G, Beilin LJ, Eguchi K, Imai Y, Kario K, Ohkubo T, Pierdomenico SD, Schwartz JE, Wing L, Verdecchia P. Contribution of the ABP-International study to the definition of night-time tachycardia. J Hypertens 2014;32:2099-2100
4. Palatini P, Reboldi G, Beilin LJ, Casiglia E, Eguchi K, Imai Y, Kario K, Ohkubo T, Pierdomenico SD, Schwartz JE, Wing L, Verdecchia P. Masked tachycardia. A predictor of adverse outcome in hypertension. J Hypertens 2017;35:487-492
5. Reboldi G, Verdecchia P, Fiorucci G, Beilin LJ, Eguchi K, Imai Y, Kario K, Ohkubo T, Pierdomenico SD, Schwartz JE, Wing L, Saladini F, Palatini P. Glomerular hyperfiltration is a predictor of adverse cardiovascular outcomes. Kidney Int 2018;93:195-203
6. Reboldi G, Verdecchia P, Saladini F, Pane M, Beilin LJ, Eguchi K, Imai Y, Kario K, Ohkubo T, Pierdomenico SD, Schwartz JE, Wing L, Palatini P. Added predictive value of high uric acid for cardiovascular events in the Ambulatory Blood Pressure International Study. J Clin Hypertens 2019;21:966-974
7. Palatini P, Verdecchia P, Beilin LJ, Eguchi K, Imai Y, Kario K, Ohkubo T, Pierdomenico SD, Saladini F, Schwartz JE, Wing L, Signorotti S Reboldi G. Association of Extreme Nocturnal Dipping With Cardiovascular Events Strongly Depends on Age. Hypertension 2020;75:324-330

**Supplementary references 6**

***Prospective Studies Collaboration***

1. Prospective Studies Collaboration. Collaborative overview ('meta-analysis') of prospective observational studies of the associations of usual blood pressure and usual cholesterol levels with common causes of death: protocol for the second cycle of the Prospective Studies Collaboration. J Cardiovasc Risk. 1999;6:315-320
2. Lewington S, Clarke R, Qizilbash N, Peto R, Collins R; Prospective Studies Collaboration. Age-specific relevance of usual blood pressure to vascular mortality: a meta-analysis of individual data for one million adults in 61 prospective studies. Lancet 2002;360:1903-1913
3. Prospective Studies Collaboration； Lewington S, Whitlock G, Clarke R, Sherliker P, Emberson J, Halsey J, Qizilbash N, Peto R, Collins R. Blood cholesterol and vascular mortality by age, sex, and blood pressure: a meta-analysis of individual data from 61 prospective studies with 55,000 vascular deaths. Lancet 2007;370:1829-1839
4. Prospective Studies Collaboration； Whitlock G, Lewington S, Sherliker P, Clarke R, Emberson J, Halsey J, Qizilbash N, Collins R, Peto R. Body-mass index and cause-specific mortality in 900 000 adults: collaborative analyses of 57 prospective studies. Lancet 2009;373:1083-1096

**Supplementary references 7**

***Chronic Kidney Disease Prognosis Consortium***

1. Chronic Kidney Disease Prognosis Consortium, Matsushita K, van der Velde M, Astor BC, Woodward M, Levey AS, de Jong PE, Coresh J, Gansevoort RT. Association of estimated glomerular filtration rate and albuminuria with all-cause and cardiovascular mortality in general population cohorts: a collaborative meta-analysis. Lancet 2010;375:2073-2081
2. Hallan SI, Matsushita K, Sang Y, Mahmoodi BK, Black C, Ishani A, Kleefstra N, Naimark D, Roderick P, Tonelli M, Wetzels JF, Astor BC, Gansevoort RT, Levin A, Wen CP, Coresh J, Chronic Kidney Disease Prognosis Consortium. Age and association of kidney measures with mortality and end-stage renal disease. JAMA 2012;308:2349-2360
3. Mahmoodi BK, Matsushita K, Woodward M, Blankestijn PJ, Cirillo M, Ohkubo T, Rossing P, Sarnak MJ, Stengel B, Yamagishi K, Yamashita K, Zhang L, Coresh J, de Jong PE, Astor BC, Chronic Kidney Disease Prognosis Consortium. Associations of kidney disease measures with mortality and end-stage renal disease in individuals with and without hypertension:a meta-analysis. Lancet 2012;380:1649-1661
4. Fox CS, Matsushita K, Woodward M, Bilo HJ, Chalmers J, Heerspink HJ, Lee BJ, Perkins RM, Rossing P, Sairenchi T, Tonelli M, Vassalotti JA, Yamagishi K, Coresh J, de Jong PE, Wen CP, Nelson RG, Chronic Kidney Disease Prognosis Consortium. Associations of kidney disease measures with mortality and end-stage renal disease in individuals with and without diabetes: a meta-analysis. Lancet 2012;380:1662-1673
5. Nitsch D, Grams M, Sang Y, Black C, Cirillo M, Djurdjev O, Iseki K, Jassal SK, Kimm H, Kronenberg F, Oien CM, Levey AS, Levin A, Woodward M, Hemmelgarn BR;,Chronic Kidney Disease Prognosis Consortium. Associations of estimated glomerular filtration rate and albuminuria with mortality and renal failure by sex: a meta-analysis. BMJ 2013;346:f324
6. Wen CP, Matsushita K, Coresh J, Iseki K, Islam M, Katz R, McClellan W, Peralta CA, Wang H, de Zeeuw D, Astor BC, Gansevoort RT, Levey AS, Levin A, Chronic Kidney Disease Prognosis Consortium. Relative risks of chronic kidney disease for mortality and end-stage renal disease across races are similar. Kidney Int 2014;86:819-827

**Supplementary references 8**

***Evidence for Cardiovascular Prevention From Observational Cohorts in Japan Research Group (EPOCH-JAPAN)***

1. Murakami Y, Hozawa A, Okamura T, Ueshima H; Evidence for Cardiovascular Prevention from Observational Cohorts in Japan Research Group (EPOCH-JAPAN). Relation of blood pressure and all-cause mortality in 180,000 Japanese participants: pooled analysis of 13 cohort studies. Hypertension 2008;51:1483-1491
2. Murakami Y, Miura K, Okamura T, Ueshima H; EPOCH-JAPAN Research Group. Population attributable numbers and fractions of deaths due to smoking: a pooled analysis of 180,000 Japanese. Prev Med 2011;52:60-65
3. Nagasawa S, Okamura T, Iso H, Tamakoshi A, Yamada M, Watanabe M, Murakami Y, Miura K, Ueshima H; Evidence for Cardiovascular Prevention from Observational Cohorts in Japan (EPOCH-JAPAN) Research Group. Relation between serum total cholesterol level and cardiovascular disease stratified by sex and age group: a pooled analysis of 65 594 individuals from 10 cohort studies in Japan. J Am Heart Assoc 2012;1:e001974
4. Asayama K, Satoh M, Murakami Y, Ohkubo T, Nagasawa SY, Tsuji I, Nakayama T, Okayama A, Miura K, Imai Y, Ueshima H, Okamura T, Evidence for Cardiovascular Prevention from Observational Cohorts in Japan (EPOCH-JAPAN) Research Group. Cardiovascular risk with and without antihypertensive drug treatment in the Japanese general population: participant-level meta-analysis. Hypertension 2014;63:1189-1197
5. Satoh M, Ohkubo T, Asayama K, Murakami Y, Sakurai M, Nakagawa H, Iso H, Okayama A, Miura K, Imai Y, Ueshima H, Okamura T, Evidence for Cardiovascular Prevention from Observational Cohorts in Japan (EPOCH–JAPAN) Research Group. Combined effect of blood pressure and total cholesterol levels on long-term risks of subtypes of cardiovascular death: evidence for cardiovascular prevention from observational cohorts in Japan. Hypertension 2015;65:517-524
6. Hirata T, Sugiyama D, Nagasawa SY, Murakami Y, Saitoh S, Okayama A, Iso H, Irie F, Sairenchi T, Miyamoto Y, Yamada M, Ishikawa S, Miura K, Ueshima H, Okamura T, Evidence for Cardiovascular Prevention from Observational Cohorts in Japan (EPOCH-JAPAN) Research Group. A pooled analysis of the association of isolated low levels of high-density lipoprotein cholesterol with cardiovascular mortality in Japan. Eur J Epidemiol 2017;32:547-557
7. Satoh M, Ohkubo T, Asayama K, Murakami Y, Sugiyama D, Yamada M, Saitoh S, Sakata K, Irie F, Sairenchi T, Ishikawa S, Kiyama M, Ohnishi H, Miura K, Imai Y, Ueshima H, Okamura T, EPOCH-JAPAN Research Group. Lifetime risk of stroke and coronary heart disease deaths according to blood pressure level: EPOCH-JAPAN (Evidence for Cardiovascular Prevention from Observational Cohorts in Japan). Hypertension 2019;73:52-59
8. Satoh M, Ohkubo T, Asayama K, Murakami Y, Sugiyama D, Waki T, Tanaka-Mizuno S, Yamada M, Saitoh S, Sakata K, Irie F, Sairenchi T, Ishikawa S, Kiyama M, Okayama A, Miura K, Imai Y, Ueshima H, Okamura T, Evidence for Cardiovascular Prevention from Observational Cohorts in Japan (EPOCH–JAPAN) Research Group. A Combination of blood pressure and total cholesterol increases the lifetime risk of coronary heart disease mortality: EPOCH–JAPAN. J Atheroscler Thromb 2021;28:6-24

**Supplementary references 9**

***Japan Arteriosclerosis Longitudinal Study (JALS)***

1. Japan Arteriosclerosis Longitudinal Study (JALS) Group. Japan Arteriosclerosis Longitudinal Study-Existing Cohorts Combine (JALS-ECC): rationale, design, and population characteristics. Circ J 2008;72:1563-1568
2. Ninomiya T, Kiyohara Y, Tokuda Y, Doi Y, Arima H, Harada A, Ohashi Y, Ueshima H, Japan Arteriosclerosis Longitudinal Study Group. Impact of kidney disease and blood pressure on the development of cardiovascular disease: an overview from the Japan Arteriosclerosis Longitudinal Study. Circulation 2008;118:2694-2701
3. Asayama K, Ohkubo T, Yoshida S, Suzuki K, Metoki H, Harada A, Murakami Y, Ohashi Y, Ueshima H, Imai Y, Japan Arteriosclerosis Longitudinal Study (JALS) group. Stroke risk and antihypertensive drug treatment in the general population: the Japan arteriosclerosis longitudinal study. J Hypertens 2009;27:357-364
4. Miura K, Nakagawa H, Ohashi Y, Harada A, Taguri M, Kushiro T, Takahashi A, Nishinaga M, Soejima H, Ueshima H, Japan Arteriosclerosis Longitudinal Study (JALS) Group. Four blood pressure indexes and the risk of stroke and myocardial infarction in Japanese men and women: a meta-analysis of 16 cohort studies. Circulation 2009;119:1892-1898
5. Tanabe N, Iso H, Okada K, Nakamura Y, Harada A, Ohashi Y, Ando T, Ueshima H, Japan Arteriosclerosis Longitudinal Study Group. Serum total and non-high-density lipoprotein cholesterol and the risk prediction of cardiovascular events - the JALS-ECC -. Circ J 2010;74:1346-1356
6. Yatsuya H, Toyoshima H, Yamagishi K, Tamakoshi K, Taguri M, Harada A, Ohashi Y, Kita Y, Naito Y, Yamada M, Tanabe N, Iso H, Ueshima H, Japan Arteriosclerosis Longitudinal Study (JALS) group. Body mass index and risk of stroke and myocardial infarction in a relatively lean population: meta-analysis of 16 Japanese cohorts using individual data. Circ Cardiovasc Qual Outcomes 2010;3:498-505
7. Asayama K, Hozawa A, Taguri M, Ohkubo T, Tabara Y, Suzuki K, Ando T, Harada A, Ohashi Y, Ueshima H, Toyoshima H, Imai Y; Japan Arteriosclerosis Longitudinal Study (JALS) group. Blood pressure, heart rate, and double product in a pooled cohort: the Japan Arteriosclerosis Longitudinal Study. J Hypertens 2017;35:1808-1815
8. Asayama K, Kinoshita Y, Watanabe S, Ohkubo T, Ando T, Harada A, Ohashi Y, Ueshima H, Imai Y; Japan Arteriosclerosis Longitudinal Study (JALS) group. Impact of diastolic blood pressure threshold for the young population: the Japan Arteriosclerosis Longitudinal Study (JALS). J Hypertens 2019;37:652-653

**Supplementary references 10**

***Fact-finding survey and awareness survey on out of clinic blood pressure measurements***

1. Ohkubo T, Obara T, Funahashi J, Kikuya M, Asayama K, Metoki H, Oikawa T, Takahashi H, Hashimoto J, Totsune K, Imai Y, J-Home Study Group. Control of blood pressure as measured at home and office, and comparison with physician's assessment of control among treated hypertensive patients in Japan: First report of the Japan home versus office blood pressure measurement evaluation (J-HOMED) study. Hypertens Res 2004;27:755-763
2. Obara T, Ohkubo T, Funahashi J, Kikuya M, Asayama K, Metoki H, Oikawa T, Hashimoto J, Totsune K, Imai Y. Isolated uncontrolled hypertension at home and in the office among treated hypertensive patients from the J-HOME study. J Hypertens 2005;23:1653-1660
3. Obara T, Ohkubo T, Kikuya M, Asayama K, Metoki H, Inoue R, Oikawa T, Murai K, Komai R, Horikawa T, Hashimoto J, Totsune K, Imai Y, J-HOME Study Group. The current status of home and office blood pressure control among hypertensive patients with diabetes mellitus: The Japan home versus office blood pressure measurement evaluation (J-HOME) study. Diabetes Res Clin Pract 2006;73:276-283
4. Oikawa T, Obara T, Ohkubo T, Kikuya M, Asayama K, Metoki H, Komai R, Murai K, Hashimoto J, Totsune K, Imai Y, J-HOME study group. Characteristics of resistant hypertension determined by self-measured blood pressure at home and office blood pressure measurements: The J-HOME study. J Hypertens 2006;24:1737-1743
5. Murai K, Obara T, Ohkubo T, Metoki H, Oikawa T, Inoue R, Horikawa T, Asayama K, Totsune K, Hashimoto J, Imai Y, J-HOME study group. Current usage of diuretics among hypertensive patients in Japan: The Japan Home versus office blood pressure measurement evaluation (J-HOME) study. Hypertens Res 2006;29:857-863
6. Komai R, Obara T, Ohkubo T, Kato T, Kikuya M, Metoki H, Inoue R, Asayama K, Hara A, Tanaka K, Gonokami K, Hashimoto J, Totsune K, Imai Y, J-Home Study Group. Factors affecting heart rate as measured at home among treated hypertensive patients: The Japan home versus office blood pressure measurement evaluation (J-Home) study. Hypertens Res 2007;30:1051-1057
7. Horikawa T, Obara T, Ohkubo T, Asayama K, Metoki H, Inoue R, Kikuya M, Hashimoto J, Totsune K, Imai Y, J-HOME study group. Difference between home and office blood pressures among treated hypertensive patients from the Japan home versus office blood pressure measurement evaluation (J-HOME) study. Hypertens Res 2008;31:1115-1123
8. Obara T, Ohkubo T, Asayama K, Kikuya M, Metoki H, Inoue R, Komai R, Murai K, Hashimoto J, Totsune K, Imai Y, J-Home Study Group. Prevalence of Masked Hypertension in Subjects Treated with Antihypertensive Drugs as Assessed by Morning versus Evening Home Blood Pressure Measurements: The J-HOME study. Clin Exp Hypertens 2008;30:277-287
9. Ito K, Obara T, Ohkubo T, Gonokami K, Shinki T, Shibamiya T, Nakashita M, Kobayashi M, Funahashi J, Hara A, Metoki H, Asayama K, Inoue R, Kikuya M, Mano N, Imai Y, J-HOME Study Group. Influence of home blood pressure measuring conditions in the evening on the morning-evening home blood pressure difference in treated hypertensive patients: The J-HOME study. Blood Press Monit 2009;14:160-165
10. Obeforebara T, Ohkubo T, Satoh M, Mano N, Imai Y. Home and Office Blood Pressure Control among Treated Hypertensive Patients in Japan: Findings from the Japan Home versus Office Blood Pressure Measurement Evaluation (J-HOME) Study. Phrmaceuticals 2010;3:419-432
11. Shibamiya T, Obara T, Ohkubo T, Shinki T, Ishikura K, Yoshida M, Satoh M, Hashimoto T, Hara A, Metoki H, Inoue R, Asayama K, Kikuya M, Imai Y, J-HOME-Elderly study group. Electrocardiographic abnormalities and home blood pressure in treated elderly hypertensive patients: Japan home versus office blood pressure measurement evaluation in the elderly (J-HOME-Elderly) study. Hypertens Res 2010;33:670-677
12. Obara T, Ohkubo T, Fukunaga H, Kobayashi M, Satoh M, Metoki H, Asayama K, Inoue R, Kikuya M, Mano N, Miyakawa M, Imai Y. Practice and awareness of physicians regarding home blood pressure measurement in Japan. Hypertens Res 2010;33:428-434
13. Kobayashi M, Obara T, Ohkubo T, Fukunaga H, Satoh M, Metoki H, Asayama K, Inoue R, Kikuya M, Mano N, Miyakawa M, Imai Y. Practice and awareness of physicians regarding casual-clinic blood pressure measurement in Japan. Hypertens Res 2010;33:960-964
14. Obara T, Ohkubo T, Tanaka K, Satoh M, Ishikura K, Kobayashi M, Metoki H, Asayama K, Kikuya M, Murai Y, Mano N, Oide S, Imai Y. Pharmacists' awareness and attitude toward blood pressure measurement at home and in the pharmacy in Japan. Clin Exp Hypertens 2012;34:447-455
15. Ishikura K, Obara T, Kato T, Kikuya M, Shibamiya T, Shinki T, Ikeda U, Kobayashi Y, Metoki H, Mano N, Kuriyama S, Ohkubo T, Imai Y, J-HOME-Morning Study Group. Associations between day-by-day variability in blood pressure measured at home and antihypertensive drugs: The J-HOME-Morning study. Clin Exp Hypertens 2012;34:297-304
16. Obara T, Ohkubo T, Ishikura K, Shibamiya T, Ikeda U, Metoki H, Kikuya M, Mano N, Kuriyama S, Imai Y, J-Home study group, the J-Home-Morning study group. Change of the management of treated hypertensive patients with or without diabetes in Japan. Clin Exp Hypertens 2013;35:79-86
17. Obara T, Kikuya M, Kobayashi Y, Ishikura K, Ikeda U, Ishikuro M, Metoki H, Mano N, Kuriyama S, Ohkubo T, Imai Y, J-Home-Morning Study Group. Associations between visit-to-visit variability in blood pressure measured in the office and antihypertensive drugs: The J-HOME-Morning study. Clin Exp Hypertens 2013;35:285-290
18. Obara T, Ubeda SRG, Ohkubo T, Matsuura H, Ishimitsu T, Takata M, Rakugi H, Imai Y. Awareness of the Japanese Society of Hypertension Guidelines for the Management of Hypertension and their use in clinical practices: 2009 survey results. Hypertens Res 2015;38:400-404

**Supplementary references 11**

***Clinical pharmacology***

1. Imai Y, Fujikura Y, Minami N, Munakata M, Hashimoto J, Sakuma H, Watanabe N, Nishiyama A, Misawa S, Sekino H, et al. Pressor effect of recombinant human erythropoietin: results of home blood pressure measurements in hemodialysis patients. Nihon Jinzo Gakkai Shi 1994;36:51-56
2. Imai Y, Sekino H, Fujikura Y, Munakata M, Minami N, Hashimoto H, Sakuma H, Watanabe N, Misawa S, Nishiyama A, Abe K. Pressor effect of recombinant human erythropoietin: Results of ambulatory blood pressure monitoring and home blood pressure measurements. Clin Exp Hypertens 1995;17:485-506
3. Imai Y, Abe K, Nishiyama A, Sekino M, Yoshinaga K. Evaluation of the antihypertensive effect of barnidipine, a dihydropyridine calcium entry blocker, as determined by the ambulatory blood pressure level averaged for 24 hours, daytime and nighttime. Am J Hypertens 1997;10:1415-1419
4. Manabe Y, Murakami T, Iwatsuki K, Narai H, Warita H, Hayashi T, Shoji M, Imai Y, Abe K. Nocturnal blood pressure dip in CADASIL. J Neurol Sci 2001;193:13-16
5. Hashimoto J, Chonan K, Aoki Y, Ugajin T, Yamaguchi J, Nishimura T, Kikuya M, Michimata M, Matsubara M, Araki T, Hozawa A, Ohkubo T, Imai Y. Therapeutic effects of evening administration of guanabenz and clonidine on morning hypertension: evaluation using home-based blood pressure measurements. J Hypertens 2003;21:805-811
6. Nishimura T, Hashimoto J, Ohkubo T, Kikuya M, Metoki H, Asayama K, Totsune K, Imai Y. Efficacy and duration of action of the four selective angiotensin II subtype 1 receptor blockers, losartan, candesartan, valsartan and telmisartan, in patients with essential hypertension determined by home blood pressure measurements. Clin Exp Hypertens 2005;２７:477-489
7. Hashimoto J, Hirayama H, Hanasawa T, Watabe D, Asayama K, Metoki H, Kikuya K, Ohkubo T, Totsune K, Imai Y. Efficacy of combination antihypertensive therapy with low-dose indapamide: assessment by blood pressure self-monitoring at home. Clin Exp Hypertens 2005;２７:331-334
8. Shibasaki T, Obara T, Ohkubo T, Hara A, Metoki H, Inoue R, Asayama K, Kikuya M, Hashimoto J, Totsune K, Imai Y. Time-dependent effects of imidapril administration in patients with morning hypertension measured as home blood pressure. Clin Exp Hypertens 2008;30:243-254
9. Gonokami K, Obara T, Kobayashi M, Katada S, Hara A, Metoki H, Asayama K, Kikuya M, Ohkubo T, Imai Y. Blood Pressure-Lowering Effect and Duration of Action of Bedtime Administration of Doxazosin Determined by Home Blood Pressure Measurement. Clin Exp Hypertens 2010;32:311-317
10. Hanazawa T, Obara T, Ogasawara K, Shinki T, Katada S, Inoue R, Metoki H, Asayama K, Kikuya M, Ohkubo T, Mano N, Imai Y. Low-dose and very low-dose spironolactone in combination therapy for essential hypertension: evaluation by self-measurement of blood pressure at home. Clin Exp Hypertens 2011;33:427-436
11. Satoh M, Obara T, Ikeda U, Kobayashi Y, Metoki H, Asayama K, Kikuya M, Ohkubo T, Imai Y. Hypotensive and heart rate-lowering effects of low-dose bisoprolol determined based on self-measured blood pressure at home. Clin Exp Hypertens 2012;34:284-289
12. Elnagar N, Satoh M, Hosaka M, Asayama K, Ishikura K, Obara T, Mano N, Ohkubo T, Imai Y. The velocity of home blood pressure reduction in response to low-dose eplerenone combined with other antihypertensive drugs determined by exponential decay function analysis. Clin Exp Hypertens 2014;36:83-91
13. Metoki H, Obara T, Asayama K, Satoh M, Hosaka M, Elnagar N, Miyawaki Y, Kojima I, Ohkubo T, Imai Y, Japan-Home versus Office Blood Pressure Measurement Evaluation – Augmentation Index Study Investigators. Differential effects of angiotensin II receptor blocker and losartan/hydrochlorothiazide combination on central blood pressure and augmentation index. Clin Exp Hypertens 2015;37:294-302
14. Hosaka M, Metoki H, Satoh M, Ohkubo T, Asayama K, Kikuya M, Inoue R, Obara T, Hirose T, Imai Y, J-HOME-CARD Study group. Randomized trial comparing the velocities of the antihypertensive effects on home blood pressure of candesartan and candesartan with hydrochlorothiazide. Hypertens Res 2015;38:701-707
15. Satoh M, Haga T, Hosaka M, Obara T, Metoki H, Murakami T, Kikuya M, Inoue R, Asayama K, Mano N, Ohkubo T, Imai Y. The velocity of antihypertensive effects of seven angiotensin II receptor blockers determined by home blood pressure measurements. J Hypertens 2016;34:1218-1223
16. Hosaka M, Inoue R, Satoh M, Watabe D, Hanazawa T, Ohkubo T, Asayama K, Obara T, Imai Y, J-HOME-ALB Study group. Effect of amlodipine, efonidipine, and trichlormethiazide on home blood pressure and upper-normal microalbuminuria assessed by casual spot urine test in essential hypertensive patients. Clin Exp Hypertens 2018;40:468-475

**Supplementary references 12**

***Atherosclerosis indices in the Ohasama study***

1. Hashimoto J, Chonan K, Aoki Y, Nishimura T, Ohkubo T, Hozawa A, Suzuki M, Matsubara M, Michimata M, Araki T, Imai Y. Pulse wave velocity and the second derivative of the finger photoplethysmogram in treated hypertensive patients: their relationship and associating factors. J Hypertens 2002;20:2415-2422
2. Kimura A, Hashimoto J, Watabe D, Takahashi H, Ohkubo T, Kikuya M, Imai Y. Patient characteristics and factors associated with inter-arm difference of blood pressure measurements in a general population in Ohasama, Japan. J Hypertens 2004;22:2277-2283
3. Hashimoto J, Watabe D, Kimura A, Takahashi H, Ohkubo T, Totsune K, Imai Y. Determinants of the second derivative of the finger phtoplethysmogram and brachial-ankle pulse-wave velocity: The Ohasama Study. Am J Hypertens 2005;18:477-485
4. Watabe D, Hashimoto J, Hatanaka R, Hanazawa T, Ohba H, Ohkubo T, Kikuya M, Totsune K, Imai Y. Electrocardiographic left ventricular hypertrophy and arterial stiffness: The Ohasama Study. Am J Hypertens 2006;19:1199-1205
5. Hashimoto J, Watabe D, Hatanaka R, Hanasawa T, Metoki H, Asayama K, Ohkubo T, Totsune K, Imai Y. Enhanced radial late systolic pressure augmentation in hypertensive patients with left ventricular hypertrophy. Am J Hypertens 2006;19:27-32
6. Hashimoto J, Imai Y, O'Rourke MF. Indices of pulse wave analysis are better predictors of left ventricular mass reduction than cuff pressure. Am J Hypertens 2007;20:378-384
7. Hashimoto J, Imai Y, O'Rouke MF. Monitoring of antihypertensive therapy for reduction in left ventricular mass. Am J Hypertens 2007;20:1229-1233
8. Shintani Y, Kikuya M, Hara A, Ohkubo T, Metoki H, Asayama K, Inoue R, Obara T, Aono Y, Hashimoto T, Hashimoto J, Totsune K, Hoshi H, Satoh H, Imai Y. Ambulatory blood pressure, blood pressure variability and the prevalence of carotid artery alteration: The Ohasama study. J Hypertens 2007;25:1704-1710
9. Hara A, Ohkubo T, Kikuya M, Shintani Y, Obara T, Metoki H, Inoue R, Asayama K, Hashimoto J, Harasawa T, Aono Y, Otani H, Tanaka K, Hashimoto J, Totsune K, Hoshi H, Satoh H, Imai Y. Detection of carotid atherosclerosis in individuals with masked hypertension and white-coat hypertension by self-measured blood pressure at home: The Ohasama Study. J Hypertens 2007;25:321-327
10. Hashimoto J, Aikawa T, Imai Y. Large artery stiffening as a link between cerebral lacunar infarction and renal albuminuira. Am J Hypertens 2008;21:1304-1309
11. Hashimoto J, Westerhof BE, Westerhof N, Imai Y, Orourke F. Different role of wave reflection magnitude and timing on left ventricular mass reduction during antihypertensive treatment. J Hypertens 2008;26:1017-1024
12. Ishikawa T, Hashimoto J, Morito R, Hanazawa T, Aikawa T, Hara A, Shintani Y, Metoki H, Inoue R, Asayama K, Kikuya M, Ohkubo T, Totsune K, Hoshi H, Satho H, Imai Y. Association of microalbuminuria with brachial-ankle pulse wave velocity: The Ohasama Study. Am J Hypertens 2008;21:413-418
13. Hashimoto T, Hara A, Ohkubo T, Kikuya M, Shintani Y, Metoki H, Inoue R, Asayama K, Kanno A, Nakashita M, Terata S, Obara T, Hirose T, Hoshi H, Totsune K, Satoh H, Imai Y. Serum magnesium, ambulatory blood pressure, and carotid artery alteration:The Ohasama study. Am J Hypertens 2010;23:1292-1298
14. Miyashita H, Aizawa A, Hashimoto J, Hirooka Y, Imai Y, Kawano Y, Kohara K, Sunagawa K, Suzuki H, Tabara Y, Takazawa K, Takenaka T, Yasuda H, Shimada K. Cross-sectional characterizaion of all classes of antihypertensives in terms of central blood pressure in Japanese Hypertensive patients. Am J Hypertens 2010;23:260-268
15. Hatanaka R, Obara T, Watabe D, Kimura A, Hanazawa T, Ohba H, Ishikawa T, Aikawa T, Hara A, Metoki H, Asayama K, Kikuya M, Ohkubo T, Totsune K, Imai Y. Individual assessment of inherent arterial stiffness using nomogram and pulse wave velocity index: The Ohasama study. Clin Exp Hypertens 2011;33:147-152
16. Kikuya M, Staessen JA, Ohkubo T, Thijs L, Asayama K, Satoh M, Hashimoto T, Hirose T, Metoki H, Obara T, Inoue R, Li Y, Dolan E, Hoshi H, Totsune K, Satoh H, Wang J-G, O'Brien E, Imai Y. How many measurements are needed to provide reliable information in terms of the ambulatory arterial stiffness index? The Ohasama study. Hypertens Res 2011;34:314-318
17. Hatanaka R, Obara T, Watabe D, Ishikawa T, Kondo T, Ishikura K, Aikawa T, Aono Y, Hara A, Metoki H, Asayama K, Kikuya M, Mano N, Ohkubo T, Izumi S-I, Imai Y. Association of arterial stiffness with silent cerebrovascular lesions: The Ohasama study. Cerebrovasc Dis 2011;31:329-337
18. Kikuya M, Ohkubo T, Satoh M, Hashimoto T, Hirose T, Metoki H, Obara T, Inoue R, Asayama K, Hoshi H, Totsune K, Satoh H, Staessen JA, Imai Y. Prognostic significance of home arterial stiffness index derived from self-measurement of blood pressure: The Ohasama Study. Am J Hypertens 2012;25:67-73
19. Metoki H, Obara T, Asayama K, Satoh M, Hosaka M, Elnagar N, Miyawaki Y, Kojima I, Ohkubo T, Imai Y, Japan-Home versus Office Blood Pressure Measurement Evaluation – Augmentation Index Study Investigators. Differential effects of angiotensin II receptor blocker and losartan/hydrochlorothiazide combination on central blood pressure and augmentation index. Clin Exp Hypertens 2015;37:294-302
20. Eguchi K, Miyashita H, Takenaka T, Tabara Y, Tomiyama H, Dohi Y, Hashimoto J, Ohkubo T, Ohta Y, Hirooka Y, Kohara K, Ito S, Kawano Y, Sunagawa K, Suzuki H, Imai Y, Kario K, Takazawa K, Yamashina A, Shimada K, ABC-J II Investigator Group. High central blood pressure is associated with incident cardiovascular events in treated hypertensives:the ABC-J II Study. Hypertens Res 2018;41:947-956
21. Bursztyn M, Kikuya M, Asayama K, Satoh M, Gavish B, Ohkubo T. Do Estimated 24-h Pulse Pressure Components Affect Outcome? The Ohasama Study. J Hypertens 2020;38:1286-1292
22. Fujita A, Hara A, Kikuya M, Asayama K, Satoh M, Asakura K, Shintani Y, Uchida S, Takatsuji Y, Murakami T, Hirose T, Tsubota-Utsugi M, Inoue R, Nomura K, Metoki H, Hozawa A, Miyazaki S, Imai Y, Ohkubo T. Blood Pressure Phenotypes Defined by Ambulatory Blood Pressure Monitoring and Carotid Artery Changes in CommunityDwelling Older Japanese Adults: The Ohasama Study. Tohoku J Exp Med 2020; 252:269-279
23. Melgarejo JD, Thijs L, Wei DM, Bursztyn M, Yang WY, Li Y, Asayama K, Hansen TW, Kikuya M, Ohkubo T, Dolan E, Stolarz-Skrzypek K, Cheng YB, Tikhonoff V, Malyutina S, Casiglia E, Lind L, Sandoya E, Filipovský J, Narkiewicz K, Gilis-Malinowska N, Kawecka-Jaszcz K, Boggia J, Wang JG, Imai Y, Verhamme P, Trenson S, Janssens S, Brien EO, Maestre GE, Gavish B, Staessen JA, Zhang ZY. Relative and Absolute Risk to Guide the Management of Pulse Pressure, an Age-Related Cardiovascular Risk Factor. Am J Hypertens 2021;34:929-938

**Supplementary references 13**

***Stroke and out of clinic BP***

1. Watanabe N, Imai Y, Nagai K, Tsuji I, Satoh H, Sakuma H, Sakuma H, Kato J, Onodera N, Yamada M, Abe F, Hisamichi S, Abe K. Nocturnal blood pressure and silent cerebrovascular lesions in elderly Japanese. Stroke 1996;27:1319-1327
2. Sakuma M, Imai Y, Tsuji I, Nagai K, Ohkubo T, Watanabe N, Sakuma H, Satoh H, Hisamichi S, Abe K. Predictive value of home blood pressure measurement in relation to stroke morbidity: a population-based pilot study in Ohasama, Japan. Hypertens Res 1997;20:167-174
3. Ohkubo T, Hozawa A, Nagai K, Kikuya M, Tsuji I, Ito S, Satoh H, Hisamichi S, Imai Y. Prediction of stroke by ambulatory blood pressure monitoring versus screening blood pressure measurements in a general population: The Ohasama study. J Hypertens 2000;18:847-854
4. Asayama K, Ohkubo T, Kikuya M, Metoki H, Hoshi H, Hashimoto J, Totsune K, Satoh H, Imai Y. Prediction of stroke by self-measurement of blood pressure at home versus casual screening blood pressure measurement in relation to the Joint National Committee 7 classification. The Ohasama study. Stroke 2004;35:2356-2361
5. Ohkubo T, Asayama K, Kikuya M, Metoki H, Obara T, Saito S, Hoshi H, Hashimoto J, Totsune K, Satoh H, Imai Y. Prediction of ischemic and haemorrhagic stroke by self-measured blood pressure at home: The Ohasama study. Blood Press Monit 2004;9:315-320
6. Asayama K, Ohkubo T, Kikuya M, Metoki H, Obara T, Hoshi H, Hashimoto J, Totsune K, Satoh H, Imai Y. Use of 2003 European Society of Hypertension-European Society of Cardiology guidelines for predicting stroke using self-measured blood pressure at home: The Ohasama study. Eur Heart J 2005;26:2026-2031
7. Asayama K, Ohkubo T, Kikuya M, Obara T, Metoki H, Inoue R, Hara A, Hirose T, Hoshi H, Hashimoto J, Totsune K, Satoh H, Imai Y. Prediction of stroke by home "morning" versus "evening" blood pressure values. The Ohasama study. Hypertension 2006;48:737-743
8. Metoki H, Ohkubo T, Kikuya M, Asayama K, Obara T, Hashimoto J, Totsune K, Hoshi H, Satoh H, Imai Y. Prognostic significance for stroke of a morning pressor surge and a nocturnal blood pressure decline. The Ohasama Study. Hypertension 2006;47:149-154
9. Inoue R, Ohkubo T, Kikuya M, Metoki H, Asayama K, Obara T, Hirose T, Hara A, Hoshi H, Hashimoto J, Totsune K, Satoh H, Kondo Y, Imai Y. Stroke risk in systolic and combined systolic and diastolic hypertension determined using ambulatory blood pressure. The Ohasama study. Am J Hypertens 2007;20:1125-1131
10. Aono Y, Ohkubo T, Kikuya M, Hara A, Kondo T, Obara T, Metoki H, Inoue R, Asayama K, Shintani Y, Hashimoto J, Totsune K, Hoshi H, Satoh H, Izumi S, Imai Y. Plasma fibrinogen, ambulatory blood pressure and silent cerebrovascular lesions. The Ohasama study. Arterioscler Thromb Vasc Biol 2007;27:963-968
11. Otani H, Kikuya M, Hara A, Terata S, Ohkubo T, Kondo T, Hirose T, Obara T, Metoki H, Inoue R, Asayama K, Kanno A, Terawaki H, Nakayama M, Totsune K, Hoshi H, Satoh H, Izumi SI, Imai Y. Association of ｋidney dysfunction with silent lacunar infarcts and white matter hyperintensity in the general population: The Ohasama study. Cerebrovasc Dis 2010;30:43-50
12. Yasui D, Ohkubo T, Kikuya M, Kanno A, Hara A, Hirose T, Obara T, Metoki H, Inoue R, Totsune K, Hoshi H, Satho H, Imai Y. Stroke risk in treated hypertension based on home blood pressure: The Ohasama study. Am J Hypertens 2010;23:508-514
13. Elnimr EM, Kondo T, Suzukamo Y, Satoh M, Oouchida Y, Hara A, Ohkubo T, Kikuya M, Hirano M, Hosokawa A, Hosokawa T, Imai Y, Izumi S-I. Association between white matter hyperintensity and lacunar infarction on MRI and subitem scores of the Japanese version of mini-mental state examination for testing cognitive decline: the ohasama study. Clin Exp Hypertens 2012;34:541-547
14. Murakami K, Asayama K, Satoh M, Hosaka M, Matsuda A, Inoue R, Tsubota-Utsugi M, Murakami T, Nomura K, Kikuya M, Metoki H, Imai Y, Ohkubo T. Home blood pressure predicts stroke incidence among older adults with impaired physical function: The Ohasama study. J Hypertens 2017;35:2395-2401
15. Murakami K, Asayama K, Satoh M, Inoue R, Tsubota-Utsugi M, Hosaka M, Matsuda A, Nomura K, Murakami T, Kikuya M, Metoki H, Imai Y, Ohkubo T. Risk factors for stroke among young-old and old-old community-dwelling adults in Japan: The Ohasama study. J Atheroscler Thromb 2017;24:290-300
16. Satoh M, Murakami T, Asayama K, Hirose T, Kikuya M, Inoue R, Tsubota-Utsugi M, Murakami K, Matsuda A, Hara A, Obara T, Kawasaki R, Nomura K, Metoki H, Node K, Imai Y, Ohkubo T. N-terminal pro-B-type natriuretic peptide is not a significant predictor of stroke incidence after 5 ears - The Ohasama study. Circ J 2018;82:2055-2062
17. Nakayama S, SatohM, Metoki H, Murakami T, Asayama K, Hara A, Hirose T, Kanno A, Inoue R, Tsubota-Utsugi M, KikuyaM, Hozawa A, Imai Y, OhkuboT. Lifetime risk of stroke stratified by chronic kidney disease and hypertension in the general Asian population: The Ohasama study. Hypertens Res 2021;44:866-873

**Suoolementary reference 14**

***Chronic kidney disease***

1. Nakayama M, Metoki H, Terawaki H, Ohkubo T, Kikuya M, Sato T, Nakayama K, Asayama K, Inoue R, Hashimoto J, Totsune K, Hoshi H, Ito S, Imai Y. Kidney dysfunction as a risk factor for first symptomatic stroke events in a general Japanese population - the Ohasama study. Nephrol Dial Transplant 2007;22:1910-1915
2. Terawaki H, Metoki H, Nakayama M, Ohkubo T, Kikuya M, Asayama K, Inoue R, Hoshi H, Ito S, Imai Y. Masked hypertension determined by self-measured blood pressure at home and chronic kidney disease in the Japanese general population: The Ohasama study. Hypertens Res 2008;31:2129-2135
3. Tanaka Y, Daida H, Imai Y, Miyauchi K, Sato Y, Hiwatari M, Kitagawa A, Kishimoto J, Yamazaki T, Kawamori R. Morning home blood pressure may be a significant marker of nephropathy in Japanese patients with type 2 diabetes: ADVANCED-J study 1. Hypertens Res 2009;32:770-774
4. Kanno A, Metoki H, Kikuya M, Terawaki H, Hara A, Hashimoto T, Asayama K, Inoue R, Shishido Y, Nakayama M, Totsune K, Ohkubo T, Imai Y. Usefulness of assessing masked and white-coat hypertension by ambulatory blood pressure monitoring for determining prevalent risk of chronic kidney disease: The Ohasama study. Hypertens Res 2010;33:1192-1198
5. Mahmoodi BK, Matsushita K, Woodward M, Blankestijn PJ, Cirillo M, Ohkubo T, Rossing P, Sarnak MJ, Stengel B, Yamagishi K, Yamashita K, Zhang L, Coresh J, de Jong PE, Astor BC; Chronic Kidney Disease Prognosis Consortium. Associations of kidney disease measures with mortality and end-stage renal disease in individuals with and without hypertension: a meta-analysis. Lancet 2012;380:1649-1661
6. Fox CS, Matsushita K, Woodward M, Bilo HJ, Chalmers J, Heerspink HJ, Lee BJ, Perkins RM, Rossing P, Sairenchi T, Tonelli M, Vassalotti JA, Yamagishi K, Coresh J, de Jong PE, Wen CP, Nelson RG; Chronic Kidney Disease Prognosis Consortium. Associations of kidney disease measures with mortality and end-stage renal disease in individuals with and without diabetes: a meta-analysis. Lancet 2012;380:1662-1673
7. Kanno A, Kikuya M, Ohkubo T, Hashimoto T, Satoh M, Hirose T, Obara T, Metoki H, Inoue R, Asayama K, Shishido Y, Hoshi H, Nakayama M, Totsune K, Satoh H, Sato H, Imai Y. Pre-hypertension as a significant predictor of chronic kidney disease in a general population: The Ohasama ｓtudy. Nephrol Dial Transplant 2012;27:3218-3223
8. Kanno A, Kikuya M, Asayama K, Satoh M, Inoue R, Hosaka M, Metoki H, Obara T, Hoshi H, Totsune K, Sato T, Taguma Y, Sato H, Imai Y, Ohkubo T. Night-time blood pressure is associated with the development of chronic kidney disease in a general population:Ｔhe Ohasama study. J Hypertens 2013;31:2410-2417
9. Blood Pressure Lowering Treatment Trialists' Collaboration, Ninomiya T, Perkovic V, Turnbull F, Neal B, Barzi F, Cass A, Baigent C, Chalmers J, Li N, Woodward M, MacMahon S. Blood pressure lowering and major cardiovascular events in people with and without chronic kidney disease:meta-analysis of randomised controlled trials. BMJ 2013;347:f5680
10. Rakugi H, Ogihara T, Umemoto S, Mathuzaki M, Matsuoka H, Shimada K, Higaki J, Ito S, Kamiya A, Suzuki H, Ohashi Y, Shimamoto K, Saruta T, Combination Therapy of Hypertension to Prevent Cardiovascular Events Trial Group. Combination therapy for hypertension in patients with CKD: a subanalysis of the Combination therapy of Hypertension to Prevent Cardiovascular Events trial. Hypertens Res 2013;36:947-958
11. Nitsch D, Grams M, Sang Y, Black C, Cirillo M, Djurdjev O, Iseki K, Jassal SK, Kimm H, Kronenberg F, Oien CM, Levey AS, Levin A, Woodward M, Hemmelgarn BR, Chronic Kidney Disease Prognosis Consortium. Associations of estimated glomerular filtration rate and albuminuria with mortality and renal failure by sex: a meta-analysis. BMJ 2013;346:f324
12. Ishikura K, Obara T, Kikuya M, Satoh M, Hosaka M, Metoki H, Nishigori H, Mano N, Nakayama M, Imai Y, Ohkubo T J-HOME-Morning Study Group. Home blood pressure level and decline in renal function among treated hypertensive patients: The J-HOME-Morning Study. Hypertens Res 2016;39:107-112
13. Satoh M, Hirose T, Nakayama S, Murakami T, Takabatake K, Asayama K, Imai Y, Ohkubo T, Mori T, Metoki H. Blood pressure and ｃhronic ｋidney ｄisease ｄtratified by ｇender and the use of antihypertensive drugs. J Am Heart Assoc 2020;9:e015592
14. Nakayama S, Satoh M, Metoki H, Murakami T, Asayama K, Hara A, Hirose T, Kanno A, Inoue R, Tsubota-Utsugi M, Kikuya M, Mori T, Hozawa A, Imai Y, Ohkubo T. Lifetime risk of stroke stratified by chronic kidney disease and hypertension in the general Asian population: the Ohasama study. Hypertens Res 2021;44:866-873

**Supplementary reference 15**

***Pathophysiology of hypertension***

1. Satoh M, Kikuya M, Hara A, Ohkubo T, Mori T, Metoki H, Utsugi MT, Hirose T, Obara T, Inoue R, Asayama K, Totsune K, Hoshi H, Satoh H, Imai Y. Aldosterone-to-renin ratio and home blood pressure in subjects with higher and lower sodium intake: The Ohasama study. Hypertens Res 2011;34:361-366
2. Satoh M, Kikuya M, Ohkubo T, Mori T, Metoki H, Hashimoto T, Hara A, Utsugi MT, Hirose T, Obara T, Inoue R, Asayama K, Kanno A, Totsune K, Hoshi H, Satoh H, Imai Y. Aldosterone-to-renin ratio and nocturnal blood pressure decline in a general population: The Ohasama study. J Hypertens 2011;29:1940-1947
3. Popov S, Silveira A, Wågsäter D, Takemori H, Oguro R, Matsumoto S, Sugimoto K, Kamide K, Hirose T, Satoh M, Metoki H, Kikuya M, Ohkubo T, Katsuya T, Rakugi H, Imai Y, Sanchez F, Leosdottir M, Syvänen A-C, Hamsten A, Melander O, Bertorello AM. Salt-inducible kinase 1 influences Na(+), K(+)-ATPase activity in vascular smooth muscle cells and associates with variations in blood pressure. J Hypertens 2011;29:2395-2403
4. Satoh M, Kikuya M, Ohkubo T, Mori T, Metoki H, Hashimoto T, Hara A, Utsugi MT, Hirose T, Obara T, Inoue R, Asayama K, Kanno A, Totsune K, Hoshi H, Satoh H, Imai Y. Aldosterone-to-renin ratio and nocturnal blood pressure decline in a general population: The Ohasama study. J Hypertens 2011;29:1940-1947
5. Satoh M, Kikuya M, Ohkubo T, Imai Y. Role of angiotensinogen and relative aldosterone excess in salt-sensitive hypertension. Hypertension 2012;59:e57
6. Satoh M, Kikuya M, Ohkubo T, Mori T, Metoki H, Hara A, Utsugi MT, Hashimoto T, Hirose T, Obara T, Inoue R, Asayama K, Kanno A, Totsune K, Hoshi H, Satoh H, Imai Y. Aldosterone-to-renin ratio as a predictor of stroke under conditions of high sodium intake: The Ohasama study. Am J Hypertens 2012;25:777-783
7. Terata S, Kikuya M, Satoh M, Ohkubo T, Hashimoto T, Hara A, Hirose T, Obara T, Metoki H, Inoue R, Asayama K, Kanno A, Totsune K, Hoshi H, Satoh H, Sato H, Imai Y. Plasma renin activity and the aldosterone-to-renin ratio are associated with the development of chronic kidney disease: The Ohasama Study. J Hypertens 2012;30:1632-1638
8. Satoh M, Hosaka M, Asayama K, Kikuya M, Inoue R, Metoki H, Utsugi MT, Hara A, Hirose T, Obara T, Mori T, Totsune K, Hoshi H, Mano N, Imai Y, Ohkubo T. Aldosterone-to-renin ratio and nocturnal blood pressure decline assessed by self-measurement of blood pressure at home: The Ohasama Study. Clin Exp Hypertens 2014;36:108-114
9. Satoh M, Kikuya M, Hosaka M, Asayama K, Inoue R, Metoki H, Tsubota-Utsugi M, Hara A, Hirose T, Obara T, Mori T, Totsune K, Hoshi H, Mano N, Imai Y, Ohkubo T. Association of aldosterone-to-renin ratio with hypertension differs by sodium intake: The Ohasama Study. Am J Hypertens 2015;28:208-215

**Supplementary references 16**

***Genetic epidemiology of Ohasama study and HOMED-BP study***

1. Takami S, Imai Y, Katsuya T, Ohkubo T, Tsuji I, Nagai K, Satoh H, Hisamichi S, Higaki J, Ogihara T. Gene polymorphism of the renin-angiotensin system associates with risk for lacunar infarction. The Ohasama Study. Am J Hypertens 2000;13:121-127
2. Ishikawa K, Imai Y, Katsuya T, Ohkubo T, Tsuji I, Nagai K, Takami S, Nakta Y, Satoh H, Hisamichi S, Higaki J, Ogihara T. Human G-protein β3 subunit variant is associated with serum potassium and total cholesterol levels but not with blood pressure. Am J Hypertens 2000;13:140-145
3. Matsubara M, Ohkubo T, Michimata M, Hozawa A, Ishikawa K, Katsuya T, Nagai K, Tsuji I, Higaki J, Araki T, Satoh H, Hisamichi S, Ito S, Imai Y. Japanese individuals do not harbor the T594M mutation but do have the P592S mutation in the C-terminus of the β-subunit of the epithelial sodium channel: The Ohasama Study. J Hypertens 2000;18:861-866
4. Asai T, Ohkubo T, Katsuya T, Higaku J, Fu Y, Fukuda M, Hozawa A, Matsubara M, Kitaoka H, Tsuji I, Araki T, Satoh H, Hisamichi S, Imai Y, Ogihara T. Endothelin-1 gene variant associates with blood pressure in obese Japanese subjects. The Ohasama study. Hypertension 2001;38:1321-1324
5. Matsubara M, Kikuya M, Ohkubo T, Metoki H, Omori F, Fujiwara T, Suzuki M, Michimata M, Hozawa A, Katsuya T, Higaki J, Tsuji I, Araki T, Ogihara T, Satoh H, Hisamichi S, Nagai K, Kitaoka H, Imai Y. Aldosterone synthase gene (CPY 11B2) C-334T polymorphism, ambulatory blood pressure and nocturnal decline in blood pressure in the general Japanese population: The Ohasama Study. J Hypertens 2001;19:2179-2184
6. Fukuda M, Ohkubo T, Katsuya T, Hozawa A, Asai T, Matsubara M, Kitaoka H, Tsuji I, Araki T, Satoh H, Higaki J, Hisamichi S, Imai Y, Ogihara T. Association of a mast cell chymase gene variant with HDL cholesterol, but not with blood pressure in the Ohasama study. Hypertens Res 2002;25:179-184
7. Sugimoto K, Hozawa A, Katsuya T, Matsubara M, Ohkubo T, Tsuji I, Motone M, Higaki J, Hisamichi S, Imai Y, Ohihara T. Alpha-aducin Gly460Trp polymorphism is associated with low renin hypertension in younger subjects in the Ohasama study. J Hypertens 2002;20:1779-1784
8. Suzuki M, Sato T, Fujiwara T, Michimata M, Araki T, Metoki H, Kikuya M, Kazama I, Hashimoto J, Hozawa A, Ohkubo T, Tsuji I, Imai Y, Matsubara M. Genetic polymorphisms in the beta-subunit of the epithelial sodium channel (βENaC) gene in the Japanese population. Clin Exp Nephrol 2002;6:130-134
9. Matsubara M, Ketoki H, Suzuki M, Fujiwara KikuyaM, Michimata M, Ohkubo T, Hozawa A, Tsuji I, Hisamichi S, Araki T, Imai Y. Genotypes of the βENaC gene have little infuluence on blood pressure level in the Japanese population. Am J Hypertens 2002;15:189-192
10. Matsubara M, Suzuki M, Fujiwara T, Kikuya M, Metoki H, Michimata M, Araki T, Kazama I, Satoh T, Hashimoto J, Hozawa A, Ohkubo T, Tsuji I, Katsuya T, Higaki J, Ogihara T, Satoh H, Imai Y. Angiotensin-converting enzyme I/D polymorphism and hypertension: The Ohasama study. J Hypertens 2002;20:1121-1126
11. Matsubara M, Metoki H, Katsuya T, Kikuya M, Suzuki M, Michimata M, Araki T, Hozawa A, Tsuji I, Ogihara T, Imai Y. T+31C polymorphism (M235T) of the angiotensinogen gene and home blood pressure in the Japanese general population: The Ohasama study. Hypertens Res 2003;26:47-52
12. Kikuya M, Sugimoto K, Katsuya T, Suzuki M, Sato T, Funahashi J, Ktoh R, Kazama I, Michimata M, Araki T, Hozawa A, Tsuji I, Ogihara T, Yanagisawa T, Imai Y, Matsubara M. A/C1166 gene polymorphism of the angiotensin II type 1 receptor (AT1) and ambulatory blood pressure: The Ohasama study. Hypertens Res 2003;26:141-145
13. Katsuya T, Sugimoto K, Hozawa A, Ohkubo T, Yamamoto K, Matsuo A, Ishikawa K, Matsubara M, Rakugi H, Tsuji I, Imai Y, Ogihara T. Genetic risk factors for cerebral infarction using data from a large-scale genetic epidemiological study: The Ohasama study. Geriatr Gerontol Int 2003;3:150-153
14. Sugimoto K, Katsuya T, Ohkubo T, Hozawa A, Yamamoto K, Matsuo A, Rakugi H, Tsuji I, Imai Y, Ogihara T. Association between angiotensin II type 1 receptor gene polymorphism and essential hypertension: The Ohasama study. Hypertens Res 2004;27:551-556
15. Matsubara M, Sato T, Nishimura T, Suzuki M, Kikuya M, Metoki H, Michimata M, Tsuji I, Ogihara T, Imai Y. CYP11B2 polymorphisms and home blood pressure in a population-based cohort in Japanese: The Ohasama study. Hypertens Res 2004;27:1-6
16. Kohara K, Tabara Y, Nakura J, Imai Y, Ohkubo T, Hata A, Soma M, Nakayama T, Umemura S, Hirawa N, Ueshima H, Kita Y, Ogihara T, Katsuya T, Yakahashi N, Tokunaga K, Miki T. Identification of hypertension/susceptibility genes and pathways by a systemic multiple candidate gene approach: the millennium genome project for hypertension. Hypertens Res 2008;31:203-212
17. Mashimo Y, Suzuki Y, Hatori K, Tabara Y, Miki T, Tokunaga K, Katsuya T, Ogihara T, Yamada M, Takahashi N, Makita Y, Nakayama T, Soma M, Hirawa N, Umemura S, Ohkubo T, Imai Y, Hata Y. Association of TNFRSF4 gene polymorphism with essential hypertension. J Hypertens 2008;26:902-913
18. Hirose T, Hashimoto M, Totsune K, Metoki H, Asayama K, Kikuya M, Sugimoto K, Katsuya T, Ohkubo T, Hashimoto J, Rakugi H, Takahashi K, Imai Y. Association of (pro) renin receptor gene polymorphism with blood pressure in Japanese men: The Ohasama study. Am J Hypertens 2009;22:294-299
19. Watanabe Y, Metoki H, Ohkubo T, Katsuya T, Tabara Y, Kikuya M, Hirose T, Sugimoto K, Asayama K, Inoue R, Hara A, Obara T, Nakura J, Kohara K, Totsune K, Ogihara T, Rakugi H, Miki T, Imai Y. Accumulation of common polymorphisms is associated with development of hypertension: a 12-year follow-up from the Ohasama study. Hypertens Res 2010;33:129-134
20. Tabara Y, Kohara K, Kita Y, Hirawa N, Katsuya T, Ohkubo T, Hiura Y, Tajima A, Morisaki T, Miyata T, Nakayama T, Takashima N, Nakura J, Kawamoto R, Takahashi N, Hata A, Soma M, Imai Y, Kokubo Y, Okamura T, Tomoike H, Iwai N, Ogihara T, Inoue I, Tokunaga K, Johnson T, Caulfield M, Munroe P; Global Blood Pressure Genetics Consortium, Umemura S, Ueshima H, Miki T. Common variants in the ATP2B1 gene are associated with susceptibility to hypertension: the Japanese Millennium Genome Project. Hypertension 2010;56:973-980
21. International Consortium for Blood Pressure Genome-wide Association Studies. Association of genetic variation with systolic and diastolic blood pressure among African Americans: the Candidate Gene Association Resource study. Hum Mol Genet. 2011;20:2273-2284
22. The International Consortium for Blood Pressure Genome-wide Association Studies. Genetic variants in novel pathways influence blood pressure and cardiovascular disease risk. Nature 2011;478:103-109
23. Millennium Genome Project for Hypertension. Hunting for genes for hypertension: the Millennium Genome Project for Hypertension. Hypertens Res 2012;35:567-573
24. Takeuchi F, Yokota M, Yamamoto K, Nakashima E, Katsuya T, Asano H, Isono M, Nabika T, Sugiyama T, Fujioka A, Awata N, Ohnaka K, Nakatochi M, Kitajima H, Rakugi H, Nakamura J, Ohkubo T, Imai Y, Shimamoto K, Yamori Y, Yamaguchi S, Kobayashi S, Takayanagi R, Ogihara T, Kato N. Genome-wide association study of coronary artery disease in the Japanese. Eur J Hum Genet 2012;20:333-340
25. Kamide K, Asayama K, Katsuya T, OhkuboT, HiroseT, Inoue R, Metoki H, Kikuya M, ObaraT, Hanada H, Thijs L, Kuznezova T, Noguchi Y, Sugimoto K, Ohishi M, Morimoto S, Nakahashi T, Takiuchi S, Ishimitsu T, Tsuchihashi T, Soma M, Higaki J, Matsuura H, Shinagawa T, Sasaguri T, Miki T, Takeda K, Shimamoto K, UenoM, Hosomi N, Kato J, Komai N, Kojima S, Sase K, Miyata T, Kawano Y, Ogihara T, Rakugi H, Staessen JA, Imai Y. Genom-wide response to antihyperttensive medication using home blood pressure measurements: a pilot study nested within the HOMED-BP study. Pharmacogenomics 2013;14:1709-1721
26. Ogata S, Kamide K, Asayama K, Tabara Y, Kawaguchi T, Satoh M, Katsuya T, Sugimoto K, Hirose T, Inoue R, Hara A, Obara T, Kikuya M, Metoki H, Matsuda F, Staessen JA, Ohkubo T, Rakugi H, Imai Y. Genome-wide association study for white coat effect in Japanese middle-aged to elderly people: HOMED-BP study. Clin Exp Hypertens 2018;40:363-369
